# “Betwixt Mine Eye and Heart a League Is Took”: The Progress of Induced Pluripotent Stem-Cell-Based Models of Dystrophin-Associated Cardiomyopathy

**DOI:** 10.3390/ijms21196997

**Published:** 2020-09-23

**Authors:** Davide Rovina, Elisa Castiglioni, Francesco Niro, Sara Mallia, Giulio Pompilio, Aoife Gowran

**Affiliations:** 1 Centro Cardiologico Monzino-IRCCS, Unit of Vascular Biology and Regenerative Medicine, 20138 Milan, Italy; davide.rovina@cardiologicomonzino.it (D.R.); elisa.castiglioni@cardiologicomonzino.it (E.C.); francesco.niro95@gmail.com (F.N.); Sara.Mallia@cardiologicomonzino.it (S.M.); giulio.pompilio@cardiologicomonzino.it (G.P.); 2 Centro Cardiologico Monzino-IRCCS, Department of Cardiac Surgery, Centro Cardiologico Monzino IRCCS, 20138 Milan, Italy; 3 Department of Clinical Sciences and Community Health, Università degli Studi di Milano, 20122 Milan, Italy

**Keywords:** induced pluripotent stem cells, cardiomyocytes, disease modeling, precision medicine, inherited cardiomyopathy, muscular dystrophies

## Abstract

The ultimate goal of precision disease modeling is to artificially recreate the disease of affected people in a highly controllable and adaptable external environment. This field has rapidly advanced which is evident from the application of patient-specific pluripotent stem-cell-derived precision therapies in numerous clinical trials aimed at a diverse set of diseases such as macular degeneration, heart disease, spinal cord injury, graft-versus-host disease, and muscular dystrophy. Despite the existence of semi-adequate treatments for tempering skeletal muscle degeneration in dystrophic patients, nonischemic cardiomyopathy remains one of the primary causes of death. Therefore, cardiovascular cells derived from muscular dystrophy patients’ induced pluripotent stem cells are well suited to mimic dystrophin-associated cardiomyopathy and hold great promise for the development of future fully effective therapies. The purpose of this article is to convey the realities of employing precision disease models of dystrophin-associated cardiomyopathy. This is achieved by discussing, as suggested in the title echoing William Shakespeare’s words, the settlements (or “leagues”) made by researchers to manage the constraints (“betwixt mine eye and heart”) distancing them from achieving a perfect precision disease model.

## 1. Introduction

The rapid adoption and universality of induced pluripotent stem cell (iPSC) technology ensured that this tool became the vanguard of applications such as disease modeling, drug screening, and cell therapy. More fundamentally, following a period of deliberation and experimentation [1], iPSCs have largely supplanted embryonic stem cells (ESCs) in terms of their relative ease of use in disease modeling. This is predominantly since iPSCs possess equivalent potentials, their use entails fewer ethical restrictions, and they are superior to ESCs as they are of an adult corporeal origin that, in theory, is matchable to a clinical history. In addition, incorporating complementary fields, such as genome editing, adds further momentum to reach the target of developing precision medicine for a spectrum of complex diseases. Although iPSCs are now widely used, their continued contribution to medical discoveries is highly dependent on the resolution of several issues that still impact the application of iPSC-derived cells. Indeed, it is important that iPSCs and their derivative cells meet specific criteria with regard to quality control (QC), safety, and efficacy. The standardization of these is becoming increasingly important [2,3,4].

Muscular dystrophy (MD) was first clinically and histologically described in the mid to late 1800s [5,6,7], later followed by the description in 1955 of a seemingly milder variant [8]. The identification of the core molecular defects, i.e., spontaneous or inherited genetic mutations responsible for MD, were gradually discovered and categorized during the latter decades of the 1900s [9,10,11,12,13]. Further advances in biomedical research methods and animal models shed much light on the pathological underpinnings of MD, which are now known to involve susceptibility of myocytes to cell-membrane microruptures, aberrant calcium handling and other aberrant intracellular signaling, mitochondrial dysfunction, anomalies in electrophysiology and excitation contraction coupling, and a predisposition to cell death [14,15,16]. Despite this wealth of knowledge, MD is still incurable, and those affected are increasingly and prematurely dying due to nonischemic cardiomyopathy [17].

In the setting of genetic cardiomyopathies, cardiomyocytes derived from iPSCs (iPSC-CMs) offer particular optimism for the discovery of innovative and targetable disease mechanisms to specifically address cardiac aspects of MD. In this review, we pragmatically assess the overall potential for iPSC-CMs to fulfil researcher’s aspirations to create a perfect precision model comparable to the quandary proposed in William Shakespeare’s Sonnet 47, as quoted in the title. In parallel, we present variations to current practices required to offset the identified shortfalls in iPSC-based models of dystrophin-associated cardiomyopathy which are similar to the “leagues” made by the sonnet’s speaker to satisfy both “heart’s and eye’s delight”.

## 2. iPSCs

In 2006, Takahashi and Yamanaka published a work that revolutionized research in the stem-cell field [18]. They reported that overexpression of merely four transcription factors by somatic murine fibroblasts generated PSCs with a gene expression profile and developmental potential equivalent to ESCs. The four transcription factors—*Oct3/4*, *Sox2*, *c-Myc*, and *Klf4*—capable of reprogramming terminally differentiated mouse embryonic or adult fibroblasts to pluripotency were identified on the basis of the hypothesis that factors essential for the maintenance of ESCs have important roles in reprogramming somatic cell nuclei. The derived cells were called induced pluripotent stem cells (iPSCs). Only one year later, two research groups independently obtained iPSCs from human somatic fibroblasts, by expressing human *OCT3/4*, *SOX2*, *c-MYC*, and *KLF4* [19] or replacing *c-MYC* and *KLF4* with *LIN28* and *NANOG* [20]. In subsequent years, different studies aimed at finding enhancers and replacement-factors demonstrated that the roles of *SOX2*, *KLF4*, and *c-MYC* can be made redundant in certain conditions [18,21,22]. Taken further, these findings can be interpreted as evidence that cell identity is more plastic than previously understood.

### 2.1. The Reprogramming Process

The efficiency of somatic cell reprogramming is low, which is mainly influenced by (i) the status of the somatic cell source, e.g., degree of founder cell proliferation, developmental potential, transcriptional activity, and epigenetic signature, (ii) the methods used to deliver reprogramming factors, and (iii) the choice of reprogramming factors [23,24,25,26]. Taking the latter first, the initial method used to deliver reprogramming genes utilizes integrating viral vectors, such as retrovirus or lentivirus, which have the advantage of effectual delivery to a wide range of cell types and initiation of durable high-level reprogramming factor expression due to the incorporation of genes within the recipient cell’s genome. However, this integrating method can cause permanent genomic modifications as a consequence of the random nature of transgene integration that carries a high risk of insertional mutagenesis and tumor formation, thus limiting the clinical applications of iPSCs derived using this method [27,28]. The use of excisable polycistronic lentiviral vectors allows the removal of inserted transgenes from the genome of established iPSCs; nevertheless, some residual sequences can remain due to excision inefficiencies, and secondary transposition is possible [29,30,31]. Inefficient silencing and reactivation of inserted transgenes can influence the differentiation potential of resulting iPSCs [29,30,32,33].

To overcome these important concerns, integration-free strategies of reprogramming were developed to obtain transgene-free iPSCs. These systems allow the transient expression of reprogramming factors by transfected cells. Thus, the chances of insertional mutations and transgene residual expression or reactivation are reduced, and the host genome remains unaltered. In spite of these methods being preferentially used in many laboratories, there are reports that nonintegrating episomal vectors are retained by iPSCs at P10, and adding further concern is the observation that episomal DNA integrates within the host genome [34]. For instance, viral nonintegrating methods allow the production of iPSCs by means of nonintegrating viruses such as adeno and Sendai viruses to deliver the Yamanaka factors. Adenovirus vectors allow transient high-level expression of exogenous genes without transferring residual transgenes [35]. However, the efficiency of reprogramming is quite low, and it was reported that adenovirus-derived iPSC cell lines contained tetraploid lines that were not observed for the retro- or lentiviral-generated iPSC cell lines [36]. The Sendai virus introduces negative-sense single-stranded RNA into the cytoplasm but not the nuclei of somatic cells; therefore, genomic insertion is circumvented [37]. Expression of exogenous genes is gradually silenced by cell division, thus avoiding transgene reactivation. In addition, other nonviral methods have been developed, including the use of minicircular DNA, plasmids, minicircles, and the expression of synthetic messenger RNA (mRNA), microRNAs (miRNAs), synthetic RNA replicons, and recombinant proteins, and exposure to small molecules [38,39,40]. Despite the increased safety of newer reprogramming methods, decreased efficiency and the requirement for repeated transfections can decrease their practical appeal.

The aforementioned factors can be used as standalone reprogramming strategies or combined with other known dedifferentiating factors. Indeed, better reprogramming efficiencies and higher-quality iPSCs can be obtained using different combinations of transcription factors, mRNAs, miRNAs, proteins, or small molecules [41,42,43,44]. These reprogramming cocktails help erase somatic cell identity and induce or maintain iPSC cell identity by different means such as (i) repressing lineage-specific gene expression promoting mesenchymal-to-epithelial transition (MET), which is promoted by bone morphogenetic proteins (BMPs) or smaller Mothers against decapentaplegic (SMAD) signaling and antagonized by activation of transforming growth factor-beta (TGF-β), amongst other molecular pathways, (ii) accelerating the cell-cycle process for instance by inhibiting p53- or p21-mediated checkpoint activation or enhancers of proliferation, (iii) reducing cell area, (iv) initiating endogenous pluripotency transcription gene expression, which reinforces the reprogrammed state independent of ectopic factor expression, and (v) modulating the chromatin remodeling complexes that induce episodic epigenetic landscaping, e.g., demethylation of pluripotency gene promoter regions [45,46,47,48]. The processes have distinctive temporal activity during the reprogramming procedure; however, the finer details are slowly being uncovered, a feat that is not helped by the notorious stochastic and inefficient nature of cell reprogramming and the heterogeneity within iPSC lines. Notwithstanding these possible detractions, since 2007, iPSC technology was rapidly adopted and evolved, making these cells currently available to almost all researchers and used in diverse research fields, including cardiac disease modeling, cardiotoxicity drug screening, and cell therapy for cardiovascular diseases [49]. Overall, all nonintegrating approaches are very relevant and promising for the clinical translation of iPSCs and their progeny. However, close monitoring of exogenous transgene residual expression, reactivation, or host genome integration is required to safeguard the future utility of iPSCs as research tools and therapeutic products.

### 2.2. Variability

One of the major problems impacting iPSC-based research and applications is line-to-line variability in their biological properties, a circumstance which is not helped by the myriad of cell sources, reprogramming methods, and cell-line maintenance procedures. This variability impacts iPSC differentiation ability, tumorigenesis potential, and altered gene expression programs depending on the particular conditions of derivation and cell culture. These points represent a critical limit for the use of iPSCs to identify disease-associated phenotypes. Normally, this entails a process whereby cells derived from patient-specific iPSCs are compared to iPSC lines from unrelated healthy donors, blood relatives, or a genome-edited isogenic corrected comparator line. Many attempts have been made to identify characteristic markers to monitor iPSC variability and predicting differentiation capacities. Among these, it was demonstrated that differences in gene expression of specific pathways might alter cellular behavior and differentiation ability in vitro [49,50,51,52].

The type of somatic cells used to obtain iPSCs influences the reprogramming process and could be one of the causes of variability. In addition to the classical use of dermal fibroblasts [19], iPSCs have been generated from peripheral blood cells [53,54,55], hematopoietic stem/progenitor cells [26,56,57], keratinocytes [58,59], melanocytes [60], mesenchymal stem cells [61], neural stem cells [62,63], astrocytes [64], hepatocytes [65], oral mucosal cells, and shed renal epithelial cells [66,67]. The efficiency, kinetics, and the cocktail of factors necessary for correct reprogramming is different for each cell type [68], with some adult cell types proving more permissive to reprogramming compared to others [23,69]. Indeed, some adult cells require just a few transcription factors, notably without the need for the oncogene c-MYC, to obtain iPSCs [56,62,63,70,71]. Taken together, this suggests that reprogramming can be easily achieved in a variety of cell types and is a process influenced by the cell context, responsiveness to pluripotency induction, and maintenance of this induced pluripotent state. A recent study demonstrated that skin fibroblasts isolated from five specific anatomical regions showed distinctive features, e.g., different reprogramming efficiencies [72]. These results indicate that not only different cells but also the same cell types isolated from different anatomical sites could influence the generation of iPSCs and the variability of the obtained reprogrammed cells and their derivatives. Dermal fibroblasts and peripheral blood mononuclear cells are by far the most commonly used cells for obtaining human iPSCs, and the reprogramming pathways have been elegantly even if not universally described. In these most frequently used cell types, comparative functional and molecular screens of the reprogramming and differentiation processes were performed [69,73,74,75,76]. The studies concluded that the tissue of origin did not affect the ability of iPSCs to differentiate along the three germinal lineages. However, evidence for skewed differentiation preferences and differences in rates of MET, retention and reactivation of exogenous pluripotency factors, and developmental cell-type-dependent reversion were uncovered.

One of the major sources of variability arises from the epigenome of founder cells. In particular, epigenetic differences caused by either incomplete reprogramming [77] or by culture conditions are of particular concern as they are difficult to control and are considered by some to be unavoidable processes of iPSC derivation [78,79]. Different studies demonstrated the presence of aberrant methylation, e.g., hypermethylation at different CpG sites in clone-specific iPSCs that persist after differentiation with a yet unknown impact on disease recapitulation [80,81]. Therefore, individual clones could have different methylation levels at different loci [82], and it was speculated that this altered epigenetic signature is responsible for the variability of iPSC cell lines and their differentiation capacities [83,84]. Indeed, Kim et al., showed that iPSCs retaining epigenetic signature of the origin cells displayed differentiation propensity to the lineage of their tissue of origin [24]. However, the influence of “epigenetic memory” is not clear since, apart from the somatic cell type, it also seems to be affected by which methods were used and what laboratory performed the reprogramming. Specifically, many different studies failed to show important changes in differentiation capacity due to the donor cell type, whereas they demonstrated that iPSCs from different people are more divergent than iPSCs reprogrammed from different somatic cells of the same donor [85,86]. Lastly, epigenetic memory seems to decrease following prolonged in vitro cell culture. Taken together, these studies suggest that epigenetic alterations and, in particular, DNA methylation have an important role in the differentiation ability of iPSCs, which could have a positive or negative effect on disease modeling [87]. Nevertheless, the origins of this epigenetic variability and its functional impact are not well understood.

In addition to the epigenetic landscape of iPSC cell lines, diverse donor genetic backgrounds could influence cell functions such as self-renewal, differentiation capability, and expression of specific genes such as receptors or transcription factors, which may have unintended consequences at all stages of precision modeling, e.g., cell reprogramming, iPSC differentiation, disease modeling, and drug screening. Indeed, this occurrence was already demonstrated in different studies showing that the transcriptomes of iPSCs obtained from different subjects were more divergent than the transcriptomes of iPSCs generated from the same person or from different somatic cells of the same donor [1,88,89,90]. In particular, differences between donor subjects’ genomes were shown to impact the majority of iPSC features, including DNA methylation, mRNA levels, protein expression, pluripotency, differentiation, and morphology [52,91]. Furthermore, different genetic backgrounds seem to have an influence on the epigenetic status of the iPSCs. Indeed, it was demonstrated that the donor genome, together with the differentiation protocol, significantly changed the methylation landscape, affecting pluripotency between different iPSC cell lines derived from different subjects [92,93]. It was demonstrated that the variability between individuals causes higher interdonor variability in gene expression of iPSC-derived cells compared to the primary cells they are intended to model [94]. Moreover, using large-scale quantitative cell morphology assays, it was highlighted that donor differences contribute up to 20% to the observed phenotypic variation among iPSCs derived from healthy subjects, suggesting that the genetic background has significant impact at different levels of cellular phenotype [91].

Another source of variability between different iPSCs, linked to the genome, is the accumulation of somatic mutations frequently observed in these cell lines [52]. These gene variants could be present in the original reprogrammed somatic cell, e.g., ultraviolet-associated mutations in dermal fibroblasts, or artificially induced during the reprogramming procedure. Interestingly, it was reported that about 50% of iPSCs obtained from skin fibroblasts showed mutations due to ultraviolet (UV) damage [95]. However, it was also suggested that reprogramming and culturing affect the selection of somatic mutations that could be advantageous within the culture conditions, for example, those related with cancer [96]. Additionally, whereas these variants accumulate during culture, it was demonstrated that about 10% of all somatic variants present in iPSCs are subclonal [95], meaning that a single iPSC cell line can be composed of different subclones with different genetic backgrounds. These mutations are frequently enriched in active promoters and linked to altered gene expression; however, they do not evolve during passaging and differentiation [95]. An important aspect that needs to be considered is that some mutations that do not alter iPSC gene expression could have an important effect on expression levels in differentiated cells. For example, mutations in cardiac-specific transcription factors or functional proteins, e.g., *NKX2.5* and cardiac troponin, respectively, could have significant effects on the phenotype of cardiomyocyte-derived iPSCs, but not on iPSCs themselves or other iPSC-derived cell types [95]. Furthermore, this could have mixed impact on the disease phenotype observed in iPSC-derived disease-relevant cell types.

Many groups reported other important sources of iPSC variability due to cell culture and maintenance conditions, including passage number, growth rate, culture medium, feeding schedule, and use of frozen cells [52,94,97,98,99]. Volpato et al. compared the transcriptomic readouts of neurons differentiated from the same iPSC cell lines using the same differentiation protocols across five distinct laboratories and determined that up to 60% of the capture variations are a consequence of the laboratory of origin [99]. Metabolism and mitochondrial dynamics could also be a source of iPSC variability. Indeed, the metabolism of PSCs and somatic cells is vastly different. PSCs are characterized by glycolytic metabolism linked to the high levels of energy needed to sustain rapid cell proliferation; conversely, differentiated cell metabolism is more flexible, where energy is formed by glycolysis and oxidative phosphorylation [100,101]. Moreover, PSCs showed lower levels of mitochondrial metabolism, marked by a reduced mitochondrion content and maturity. In particular, ESC mitochondria are globular with fewer cristae and contain matrices with low electron density [100,101,102]. Thus, when somatic cells enter in the reprogramming process, together with epigenetic and transcriptional reorganization, they undergo a remodeling of metabolic processes, shifting from highly oxidative respiratory metabolism to a glycolytic state. This metabolic switch is one of the first changes that appears during somatic cell reprogramming and is required to satisfy the energy needed for survival and the process of “becoming” pluripotent [103]. In parallel to the metabolic pathway switch, reorganization of mitochondrial content is also observed. In particular, mitochondrial respiratory complexes are downregulated and mitochondrial DNA copy number and mitochondrial density are decreased, causing functional and structural changes to mitochondria [102,104,105]. However, these mitochondrial alterations are not always complete. Indeed, ultrastructural analyses of iPSCs demonstrated the presence of a mixture of mature (somatic) and immature (ESC) mitochondria, suggesting retention of metabolic memory from the original cells [51]. ESCs are able to change glucose uptake in response to the levels of oxygen, and this is essential for normal development and cellular differentiation [106,107]. iPSCs were found to be unresponsive to oxygen variations, similar to their somatic cells of origin. This aspect could affect downstream differentiation processes [108] and quite possibly disease modeling readouts. Mutations in mitochondrial DNA could also be another source of iPSC variability affecting cell metabolism [109]. Metabolomic profiling performed on iPSCs and ESCs at different passages demonstrated that early-passage iPSCs were more divergent from ESCs (about 5% of difference on 5000 metabolites). However, following extended passaging, these differences were lost, arriving to merely 0.23% difference [110]. This suggests that the culture of iPSCs increases metabolic switch, making iPSCs more similar to ESCs, thus reducing the variability between these types of PSCs.

Taken together, these sources of variability could have a significant impact on the readouts from experiments involving iPSCs or their derived cells, influencing many different aspects, including the differentiation potential and/or disease modeling recapitulation. Indeed, the obtainment, culture, and differentiation into target cells of iPSCs is a multistep procedure, where small variations at each step can accumulate, causing significantly different outcomes [111]. Experimental replication of iPSC-based models is counteracted by the variability of iPSCs and derivative cells, possibly generating technical artefacts that obscure the aspect of interest [99]. Different ways have been proposed to circumvent these drawbacks including: large stem cell biobanks, use of reference iPSC cell lines as controls or disease comparators, genome engineering to obtain isogenic controls, or insertion of disease mutation in a non-affected iPSC cell line [52]. Different consortia have created many large-scale biobanks, and the iPSCs obtained have been made available to the research community. The use of these cell-line collections have the advantage that all the cell lines meet high QC standards and their functionality is characterized in detail [112,113,114,115,116]. Another option is the selection of reference iPSC cell lines to be shared across laboratories and used in every experiment. These cell lines would become the reference point for comparisons between studies, allowing the detection of experimental variations [52]. Finally, an extensively used strategy to overcome the influence of the genetic background in the case of a known genetic disease is the use of genome engineering techniques, particularly CRISPR/Cas9 (clustered regularly interspaced short palindromic repeats/CRISPR-associated protein 9) to obtain isogenic cell lines [117]. CRISPR/Cas9 is based on the production of site-specific double-stranded DNA breaks (DSBs) that are preferentially recovered by the error-prone non-homologous end-joining (NHEJ) pathway that causes random insertions and/or deletions of nucleotides. Alternatively, the homology-directed repair (HDR) pathway can act in proliferating cells by repairing the DSB using a wild-type (WT) sequence of the gene or a supplied exogenous DNA molecule as a template, leading to correction of the mutant allele [118,119]. These methods allow the modification of a specific site of the genome in order to “correct” disease-causing mutations in patient-specific iPSCs. On the other hand, they can also be used to introduce specific mutations into WT iPSCs in an attempt to replicate the disease. Isogenic cell lines are derived from the same subject but are engineered to differ at only one specific locus while the other loci remain identical. The use of isogenic iPSCs excludes the effect of genetic background, enabling the analyses of phenotypic impact of a confirmed single mutation [49].

### 2.3. iPSCs versus ESCs

ESCs were the first PSCs investigated and used for in vitro experiments, and they are considered the “gold standard” of PSCs [120]. These PSCs are derived from the internal cell bulk of blastocysts of the inner cell mass in preimplantation embryos. ESCs are able to differentiate into virtually all cells of the three germ layers (ectoderm, mesoderm, and endoderm). The principal biological characteristics of iPSCs are very similar to ESCs in terms of their morphology, proliferation, epigenetic patterns, telomerase activity, pluripotent gene expression, and surface antigens. Indeed, comparisons of transcriptional profiles, epigenetic status, and differentiation potential confirmed the resemblance between iPSCs and ESCs. In addition, germ line transmission was shown by generating viable offspring in the stringent tetraploid complementation assay; however, this is limited to murine iPSCs [27,121,122,123,124], since distinguishing genuine human iPSCs in this manner is not possible due to obvious ethical issues. However, the benefit of expending such a high degree of effort to compare the similarities between these two artificial derivatives in order to declare which one is the gold standard is of questionable value [125], although differences between the two types of PSCs have been reported and controversies about the extent and importance of these dissimilarities have not been clarified [126]. For instance, iPSCs show a lower developmental potential and differentiation ability compared to ESCs, depending on various initial states of pluripotency [50]. As discussed in the previous section, other influential factors are the conditions of cell-line maintenance, epigenetic status, and capacity to produce intracellular growth factors [39]. Indeed, initial studies suggested that the reprogramming procedure could cause altered epigenetic behavior in iPSCs with respect to ESC [80,81]. Nevertheless, a later study analyzed this issue in isogenic human ESCs and iPSCs, demonstrating that differences in transcriptional profile between iPSCs and ESCs are inconsistent, and, for all practical purposes, these two types of PSCs are molecularly and functionally equivalent [1]. This may explain, to some extent, the ambiguity in the conclusions from studies that assigned variations in iPSCs to differences in genetic background, reprogramming methods, and culture conditions [68].

Notwithstanding the very close similarity between ESCs and iPSCs, in the last few years, the majority of research focused on the use of iPSCs as the result of a series of ethical and technical issues and disease recapitulation potential. With regard to ethical problems, ESC isolation requires embryo destruction that makes their use problematic for those who morally consider the embryo as equivalent to a potential physical being [127]. Therefore, the essential question posed is whether it is morally acceptable to destroy an early human embryo to obtain human ESC cell lines that could be used for in vitro studies or for novel therapies [128]. This ethical quandary involving individual moral/political beliefs did not allow the development of a unique policy acceptable to everyone. Indeed, this issue has led to different legislation throughout the world [127,128]. In addition to the ethical concerns, the therapeutic applications of ESCs are limited by issues that include survival and efficacy of delivered cells, immune rejection of allogeneic grafts, and oncogenic risk. However, some concerns also regard the in vitro applications of ESCs. As reported above, to make an ESC cell line, it is necessary to destroy an embryo; thus, the cell line does not match with any living being. This aspect limits the benefits of using ESCs for disease modeling and precision medicine, because the health of the potential individual represented by the source embryo is unknowable for most diseases. In addition, the majority of embryos utilized to obtain the ESCs are donated remnants from fertility treatments and may not carry any strong predisposition to any particular disease [49]. Overall, on balance, iPSCs are more promising for both therapeutic and research purposes since they are associated with fewer ethical and technical issues [129]. However, it would be erroneous to consider iPSCs free from ethical considerations and regulations.

### 2.4. iPSC Applications

Due to their ability to differentiate into any type of cell, iPSCs have found three main applications: disease modeling, drug screening, and cell therapies.

#### 2.4.1. Disease Modeling

Since inception, iPSC technology has shown enormous potential to model disease, solving many challenges associated with traditional approaches such as animal and primary cell/tissue models. On the basis of their characteristics, patient-specific iPSCs can provide disease-related cells which may have been previously inaccessible, e.g., neurons and cardiomyocytes. Taking advantage of these intrinsic properties, iPSCs carrying patient-specific mutations can be used to model the molecular mechanisms underlying the disease pathophysiology and screen responses to various types of therapeutics. The phenotype ranges that can be investigated by iPSC models involve a broad range of molecular, metabolic, electrophysiological, and cellular analytic techniques. iPSC disease models have been widely applied to study monogenic disorders that are caused by a single gene mutation [130] and sporadic complex disorders involving multiple or unknown genes [131]. The use of iPSC-based models for the latter disease type is more problematic with respect to monogenic diseases, since the phenotype is often the result of multiple small-effect genetic variants in combination with environmental factors. However, this approach was used to model many different complex diseases including Alzheimer’s disease, Parkinson’s disease, schizophrenia, and cardiac arrhythmias [132,133,134,135]. Without knowing the detailed underlying genetics, differentiated patient-specific iPSCs could provide disease-relevant cells that carry all the genetic elements implicated in the development of the disease and can be useful to analyze the common mechanisms of disease development. Indeed, patient-specific iPSCs obtained from multiple affected individuals that show similar phenotypes could be comparatively investigated in order to find common altered mechanistic pathways or functional activities.

One of the major issues concerning disease modeling using iPSCs is the relative immaturity of the cells differentiated from iPSCs. On the basis of this observation, iPSC-based models are considered more suitable for disorders with an early onset rather than late onset, for which cellular aging could play a role in the disease phenotype. However, despite their fetal phenotype, iPSC-derived cells have highlighted different phenotypes, suggesting that the pathology starts before the appearance of clinical symptoms, potentially allowing the discovery of novel mechanisms involved in the development of pathology [52,136].

Recently, in order to better model disease phenotype, researchers have moved from single-cell culture to coculture of different cell types relevant to the specific disease being modeled. Indeed, for many diseases, more than one type of cell is involved in the development of the phenotype, and the interactions between these different cells appear to play an important role. For these reasons, the coculture of different cell types has been shown to result in a better model of the disease in which it is possible to study non-cell-autonomous aspects, including the effect of one cell type on other disease-relevant cells [137]. In addition, these complex models were also shown to increase the cell maturity of iPSC-derived cells [137,138].

Lastly, the question of what the best appropriate and adequate type of control is to include in experiments is of crucial importance. In initial studies, the iPSCs reprogrammed from cells obtained from healthy individuals or first-degree relatives were used as controls to compare patient-derived iPSCs. As described earlier, line-to-line iPSC variations and heterogeneity in iPSCs from distinct donors carrying the same genotype complicates data interpretation and the ability to discriminate line-to-line versus donor-to-donor variations from actual disease-related phenotypes. This problem is partially overcome by the development of genome editing technologies that provide genetically matched isogenic iPSCs, ensuring the detection of the true disease-phenotype variances, thus avoiding any disparities caused by genomic background or epiphenomena.

#### 2.4.2. Drug Screening

iPSC-derived cells and disease models involving iPSCs have been widely exploited in drug screening and repositioning studies. The important requirements for a phenotype screen are strong and simple-to-analyze disease-related readouts or endpoints, e.g., approaches used to define cellular responses resulting from cardiac and neurological dysfunction in different rare diseases [139,140,141]. Additionally, iPSC platforms have been employed for target-based drug screening. Indeed, differentiation into specific somatic cells allows testing the targeted effects in disease-related cells which is of crucial importance for precision efficacy. An important aspect in the development of new drugs is the evaluation of undesired effects and toxicity. In fact, many unexpected effects of new molecules can occur, with hepatic and cardiac toxicity being of particular concern. On this basis, the use of iPSC-derived cells can be useful to predict potential side effects, enabling the selection of candidate drugs that are less likely to fail owing to toxicity in late stage trials or, more seriously, in the post-authorization stage of the product lifecycle. Cardiotoxicity is one of the major causes for drug withdrawals; for this reason, iPSC-CMs can be used to screen for drug-induced alterations in cardiac cellular contractility, electrophysiology, and viability. Different studies showed that iPSC-CMs can be a powerful and sensitive tool to test drug-induced arrhythmias [142,143,144]. Moreover, it is possible to use iPSC-CMs from patients with different hereditary cardiac diseases, e.g., cardiomyopathy associated with mutations in the dystrophin gene, or to study different responses elicited by specific drugs versus iPSC-CMs from healthy subjects or genome-edited isogenic controls. These approaches are useful to predict the effectiveness of specific molecules in patients with different genetic backgrounds [145]. Indeed, since each iPSC cell line maintains the genome of the patient, iPSC-derived cells could be used to perform pharmacogenetic studies. Such “clinical trials in a dish” could be extremely useful to find the better drug for each patient group and to detect the specific responses to particular molecules [146]. For example, Burridge et al. demonstrated that iPSC-CMs obtained from breast cancer patients who developed doxorubicin-induced cardiotoxicity recapitulated this phenotype in vitro [147]. This work highlighted that iPSC-CMs can be used as a platform for predicting the phenotypic impact of a potential cardiotoxic treatment, predicting the severity of this toxicity and to identify the pharmacogenomic mechanisms [147].

#### 2.4.3. Cell Therapies

In addition to disease modeling and drug screening, iPSCs are considered one of the more promising sources of personalized cells for regenerative therapies. The ideal goal here is that iPSCs generated from the patient requiring treatment can be differentiated ex vivo to the somatic cells affected by the disease (with or without the use of gene therapy to correct the genetic defect if present) that are finally transplanted into the patient to have an ameliorating impact on the disease. However, this approach is considered a long-term application because there are different obstacles for the safe application of iPSC-based cell therapy in humans. In the first instance, one concern is the tumorigenicity risk of iPSCs and iPSC-derived cells contaminated with undifferentiated iPSCs. Some of the reprogramming factors used in iPSC generation (in particular c-MYC) are known proto-oncogenes. Moreover, the use of integrating vectors to overexpress the reprogramming factors can alter the cell genome. Another important concern is the complex culture conditions used to generate, maintain, and differentiate iPSCs that can impact the cells, e.g., maintenance in culture for prolonged periods can cause the accumulation of genomic abnormalities, copy-number variations and the loss of heterozygosity. For these reasons, differentiated cells derived from iPSCs need to be screened for the lack of potentially risky genetic abnormalities. iPSCs with such abnormalities or those produced using the overexpression of c-MYC can form teratomas. While, to date, well-characterized and homogeneous populations of cells differentiated from iPSCs do not show overt signs of tumor formation, it is still fundamental to guarantee that the final product is free from undifferentiated or partially differentiated cells and cells containing genetic aberrations that could maintain the potential to produce teratomas. In addition to these issues, compliance with good manufacturing practice is mandatory before the wide-scale transplantation of iPSC-based cell therapy products in humans, e.g., it is imperative to use xeno-free components for all procedures starting from reprogramming through to differentiation, extended to patient delivery, particularly in cases where transplanted cells are incorporated in an extracellular support matrix. However, despite these challenges, the use of iPSC-derived cells in regenerative medicine is continuously progressing, and many different iPSC-derived cells have been used in clinical trials to treat different diseases including ophthalmic, neurological, and cardiac disorders [131].

## 3. Muscular Dystrophy (MD)

**Figure 1:**
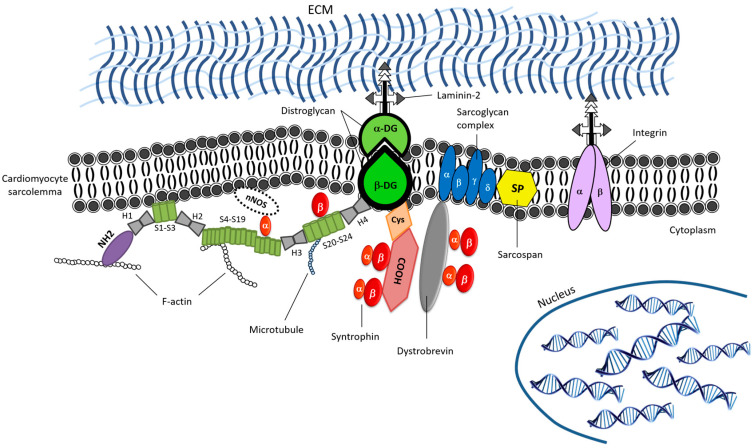
Schematic representation of the dystrophin glycoprotein complex (DGC). The dystrophin glycoprotein complex (DGC) is composed of four different sections according to their localization on the plasma membrane: (i) α-dystroglycan on the extracellular surface acts as a receptor for the intermediate filament laminin that works together with the DGC to maintain cell-basal lamina adhesion; (ii) in the transmembrane region, α-dystroglycan binds to β-dystroglycan and sarcoglycan proteins (α, β, γ, δ); (iii) within the sarcolemma, sarcospan joins the sarcoglycan complex to integrin proteins; (iv) β-dystroglycan and dystrophin anchor the sarcolemma to the intracellular domain of the DGC, which stabilizes the contractile apparatus of myocytes and the remaining part of the DGC via binding to the actin network. Crucially, as a whole entity, the DGC secures the correct location of neuronal nitric oxide synthase (nNOS), an essential enzyme that produces nitric oxide (NO), which is required to modulate vascular tone among other essential cellular signaling needed to meet tissue demands.

Muscular dystrophies are a broad grouping of more than 30 degenerative neuromuscular diseases characterized by progressive functional and structural deterioration and subsequent impairment of facial, limb, respiratory, and cardiac muscles [148]. The majority of these syndromes are caused by mutations in the genes that encode several proteins belonging to the dystrophin glycoprotein complex (DGC) or sarcomeric and other proteins that play a role in stabilizing several cell functions, e.g., appropriate ion-channel activity and stability of the sarcolemma. For the unfamiliar and on first consideration, the DGC may appear to be quite a jumble of proteins, with similar sounding names that have a wide array of individual specialized functions, e.g., dystrophin, syntrophins, dystroglycans, sarcoglycans, sarcospans, dystrobrevins, caveolin-3, and nitric oxide (NO) synthase (see Figure 1) [149,150,151]. The DGC has many functions ranging from maintenance of membrane stability and integrity to linking the intracellular structures such as the actin cytoskeleton and extracellular matrix (ECM) components, as well as orchestrating certain cell signaling pathways [152].

Among MD syndromes, mutations in the gene encoding the dystrophin protein, which is also called the *dystrophin* gene or the *DMD* gene (*Duchenne muscular dystrophin*), cause the most common forms of MD and are the specific focus of this review. There are two types of MD associated with *DMD* mutations: Duchenne’s muscular dystrophy (DMD) and Becker’s muscular dystrophy (BMD). Broadly speaking, DMD affects an estimated 1/3500 male births and shows the most severe phenotype, usually with an early symptom onset (3–5 years old) that in most cases rapidly progresses to loss of ambulation by 7–12 years of age or younger and premature death by the second or third decade of life, commonly due to heart failure (HF). DMD often coincides with lordosis, scoliosis, and low intelligence quotient (IQ). In contrast, BMD has a lower incidence than DMD (1/11,500 to 1/19,000 male births) [153] and frequently presents with a milder but more variable clinical phenotype. Muscle weakness appears later in childhood or in adolescence and generally progresses at a slower rate with loss of ambulation occurring within the second or third decade of life. However, premature death by the fourth decade is also a clinical reality for individuals with BMD [154]. In addition to close clinical observations, family medical anamnesis and monitoring serum creatine kinase (CK), DMD, and BMD are traditionally conclusively diagnosed by evaluating skeletal muscle biopsies for the localization and abundance of dystrophin protein expression, variation in myofiber size, myocyte necrosis, macrophage and other immune-cell infiltration, and fibrous-adipose replacement, although the latter invasive diagnostic procedure is steadily being replaced by detailed molecular genetic testing [155,156].

### 3.1. Dystrophin Gene and Protein

**Figure 2:**
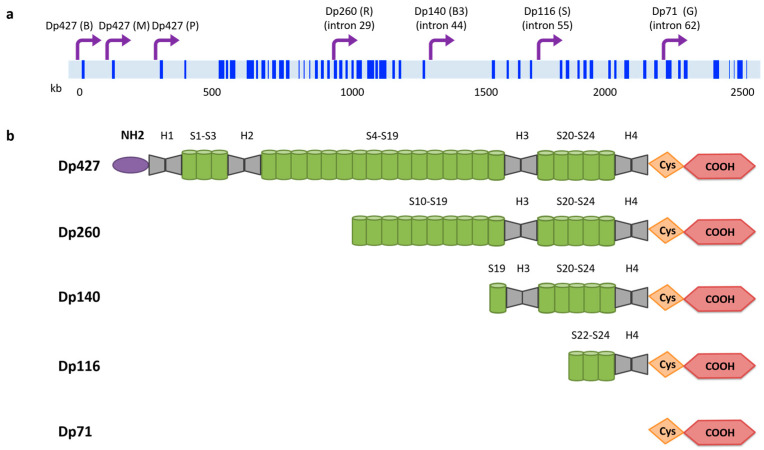
Schematic representation of the dystrophin gene and protein. (**a**) Linear representation of the *DMD* (*Duchenne muscular dystrophin*) gene. The location of the isoform-specific promoters (brain (B), muscle (M), Purkinje (P), retinal (R), brain-3 (B3), Schwann cell (S), and general (G)) is highlighted by violet arrows. Vertical blue bars indicate exons. (**b**) Structure of the different isoforms codified by the *DMD* gene. The full-length dystrophin represented consists of the following functional domains: (i) amino-terminal actin-binding domain (ABD1) that binds F-actin; (ii) central rod domain that includes a second actin-binding domain (ABD2), 24 spectrin repeats (SR1-24), and four flexible proline-rich spacer hinge regions (H) that confer elasticity and permit linkage to β-dystroglycan; (iii) cysteine-rich (CR) domain that contains two EF-hand and zinc finger motifs (ZZ) which respectively bind β-dystroglycan and calmodulin; (iv) carboxyl-terminus containing cysteine-rich and dystroglycan-interacting domains which provide binding sites for dystrobrevin and the syntrophins. The shorter isoforms lack the N-terminal domain and partially the road domain. Dp71 comprises only the CR and C-terminal domains.

*DMD* is localized on the short arm of the X chromosome (Xp21.1–21.2). Its length of >2200 kb represents one of the largest portions of the human genome and accounts for 0.1% of the entire genome. *DMD* contains 79 exons that correspond to 0.6% of the *DMD* gene, indicating that the vast majority of the gene is nonprotein-coding introns [157,158]. The *DMD* gene encodes many dystrophin protein isoforms depending on which promoter is used to initiate transcription (Figure 2a). The full-length messenger RNA (mRNA) that encodes the 427 kDa dystrophin protein can be transcribed from three different promoters, named in accordance to the tissue where they are predominantly expressed: brain (B), muscle (M; both cardiac and skeletal muscle), and Purkinje cells (P). Additional *DMD* promoters allow the transcription of shorter dystrophin isoforms in other specific tissues: retinal (R, Dp260), brain-specific (B3, Dp140), Schwann cells (S, Dp116), and the general isoform (G, Dp71). Finally, alternative splicing of *DMD* mRNA produces other dystrophin isoforms. Overall, the complexity of *DMD* transcription and translation suggests a functional diversity for dystrophin proteins of currently unknown functional significance [159,160,161].

The full-length dystrophin protein (Dp427) is a rod-shaped protein that consists of about 3685 amino acids with a molecular weight of 427 kDa. This large protein contains four different protein domains: the amino-terminal domain, the central rod-like domain, the cysteine-rich domain, and the C-terminal domain. Dp427 localizes underneath the membrane of both skeletal and cardiac myocytes, and interacts with the actin cytoskeleton and the DGC, thus establishing a bridge between the intracellular cytoskeleton and the ECM. On the other hand, shorter dystrophin isoforms lack the amino-terminal actin-binding domain but maintain a part of the rod domain (with the exception of Dp71), as well as the cysteine-rich domain and the carboxy-terminal domain, which present the binding sites for dystroglycan, dystrobrevin, and syntrophin (Figure 2b). The functions of these shorter dystrophin proteins, similar to their expression patterns, seem to be tissue-specific [159,162].

### 3.2. DMD Mutations

Due to the large size and intricate regulation, the *DMD* gene is prone to spontaneous or inherited mutations. De novo mutations are estimated to be responsible for 10–30% of cases [156,163]. Regarding the types of *DMD* mutations, the most common categories, in descending order, are as follows: deletions of one or multiple exons (65–72%) which center around the hotspot region spanning exons 45–53; duplications in one or multiple exons (6–10%) which also tend to group in a second hotspot region involving exons 2–20; mutations in noncoding regions and small gene variations, e.g., point mutations, indels, or chromosomal inversions/rearrangements, which represent the remaining 20% of *DMD* mutations [156,159,162,164,165]. Both deletions and duplications are concentrated between exons 44 and 55 (breakpoint in intron 44) [156] and between exons 3 and 19 (breakpoint in intron 2 or 7). The impact of *DMD* mutations depends on the deletion size, the location within crucial regions of the dystrophin protein, and the stability of generated mutant proteins.

The issue of phenotype difference and prediction was, in the past, explained by the “reading frame rule”, proposed by Monaco et al. (1988) [166]. They suggested that if a *DMD* mutation disrupts the open reading frame (ORF), the resulting dystrophin protein will be absent, which typically leads to a severe phenotype, i.e., DMD. On the contrary, if the ORF is not compromised by the *DMD* mutation, the expression of a partial transcript of the dystrophin protein is possible, resulting in the presence of a partially functional protein, and is usually associated with the milder BMD phenotype [166]. However, shorter or longer rod domains, as well as some truncated and partially functional dystrophin proteins, conserve the N- and C-terminal regions that are crucial for the connection of actin with the ECM. Thus, partial function can be maintained. On the other hand, frameshift mutations cause an unstable mRNA *DMD* transcript that leads to the production of low levels of unstable truncated proteins. The reading frame hypothesis holds for about the 90% of DMD/BMD cases; however, there are multiple examples where this model does not fit the clinically observed disease phenotype [167]. For example, patients carrying a frameshift mutation such as deletion of exons 3–7 have been diagnosed with BMD rather than DMD. This is normally a consequence of exon skipping events that allow the restoration of the frame with the production of truncated dystrophin [159]. On the other hand, some in-frame mutations are associated with the DMD phenotype. This is frequent for large deletions in the 5′ region that extend into the rod domains [168].

### 3.3. Pathomechanisms Associated with Dystrophin Protein Deficiency

Dystrophin acts as a myocyte stabilizer linking contractile myofibrils to the sarcolemma that ensures force transduction during muscle contraction. It is also involved in coordinating many signaling pathways which include neuronal nitric oxide synthase (nNOS) signaling, extracellular-mediated signals to mitogen-activated protein kinase (MAPK) and Ras-related C3 botulinum toxin substrate 1 (Rac1), activity of ion transporters, e.g., voltage-gated sodium channels and transient receptor potential cation (TRPC) channels, and the ligation and activation of G protein-coupled receptor signaling [169]. Dystrophin absence or deficiency impairs cell stability leading to sarcolemma microtears, DGC destabilization, and dysregulation of many molecular mechanisms closely dependent on functional dystrophin protein, including calcium homeostasis.

Intracellular calcium (_i_Ca^2+^) plays a fundamental role in maintaining many cellular processes. The concentration of _i_Ca^2+^ is finely regulated by the complex activity and interactions among calcium channels, calcium-binding proteins, and voltage-sensitive ion pumps and exchangers [170]. Calcium levels in dystrophin-deficient fibers are considerably increased due to the strong influx of extracellular calcium that triggers protein effectors such as calmodulin calcium-dependent kinase II (CamKII) and phosphoinositide 3-kinases (PI3Ks) causing further calcium release from the sarcoplasmic reticulum (SR) [171,172]. Furthermore, plasma-membrane instability leads to a pathological rearrangement of DGC-associated proteins that can be directed for intracellular proteolysis by the ubiquitin-proteasome system. This internalization process plays a central role in the activation of nuclear factor kappa-light-chain-enhancer of activated B cells (NF-κB) [173], a transcription factor required for proinflammatory gene expression, such as inducible NOS (iNOS) that reduces the activity of sarco-endoplasmic reticulum calcium ATPase (SERCA) [174] and destabilizes ryanodine receptors (RyRs), two important elements involved in calcium SR regulation, leading to SR calcium depletion [175]. Lastly, activity of the calcium-activated protease, calpain, is dysregulated in the absence of dystrophin, which results in further excessive stimulation of calcium SR release and initiates cardiac troponin degradation and release into the circulation [176,177]. Dystrophin deficiency is the main cause of endothelial NOS/nNOS delocalization, which in turn decreases NO levels causing reduced blood vessel dilation and oxygen consumption that both contribute to cell and tissue function impairment [178]. Additional downstream pathomechanisms can involve mitochondrial permeability transition pore (mPTP) opening, overproduction of reactive oxygen species (ROS), cytochrome C release, DNA damage, and lipid peroxidation. Increased levels of ROS causes damage to cellular proteins and membranes; moreover, ROS also activate NF-κB pathways that lead to increased levels of proinflammatory cytokines [179,180]. This proinflammatory signaling is deleterious for myocytes and causes cell damage and necrosis.

In skeletal muscle, the first stage of MD disease is characterized by replacement of necrotic myofibers by skeletal muscle stem cells (satellite cells) which are stimulated to enter the cell cycle, proliferate, fuse together, and repair and replace damage skeletal muscle tissue. However, satellite cells lacking dystrophin are unable to sustain the high regeneration rates demanded in the MD context, resulting in the loss of regenerative capacity and instigation of fibro-fatty tissue substitution instead [181]. In parallel, TGF-β is constitutively activated, which negatively regulates cell differentiation and regeneration by inducing continuous connective tissue fibrosis via the heterogeneous SMAD profibrotic pathway [182,183], TGF-β superfamily members, e.g., myostatin, connective-tissue growth factors, e.g., osteopontin, and ECM-degrading enzymes e.g., tissue inhibitor of metalloproteinases-1 [184].

### 3.4. Dystrophin-Associated Cardiomyopathy

Reduced respiratory-related deaths due to new therapeutic approaches, e.g., nocturnal ventilation and spinal stabilization, have increased the lifespan of people with MD. However, over time, cardiac muscle develops defects caused by dystrophin deficiencies. Therefore, the number of MD patients with cardiomyopathy is rising, and it has become the leading cause of premature death. Following a long subclinical stage, dystrophin-associated cardiomyopathy (DAC) mainly presents as dilated cardiomyopathy, although there have been many reports of hypertrophied, noncompaction, or restrictive presentations of DAC. Arrhythmias, of all types, frequently complicate DAC, e.g., sinus tachycardia preceding systolic dysfunction occurs early in the disease course; however, this is not considered as a predictor of DAC severity [185]. Additionally, DAC often manifests in 10–60% of females carrying a *DMD* mutation, who, in many instances, do not develop skeletal muscle pathology [186,187]. No strong genotype–phenotype correlations exist, and no large-scale investigations accurately predict if DAC will occur or what the likely rate of disease progression is. Statistical analysis revealed that deletions in the N-terminal domain of *DMD* (deletions in exon 1/intronic deletions/deletions in *DMD* promoter regions B, M, and P) are usually associated with an early-onset severe cardiac phenotype. On the contrary, patients with rod-domain deletions exhibit a later-onset cardiac phenotype [188]. Over 80% of DMD patients develop cardiac dysfunction by 18 years of age, while 75% BMD patients usually show cardiac symptoms by their fourth decade of life, although 15% of BMD cases exhibit DAC symptoms before 16 years of age [185].

The natural course of DAC can be divided in three pivotal phases: (i) a preclinical phase of disease, when patients have no HF symptoms; (ii) a transitional phase of the disease with symptoms and detectable signs of cardiac impairment being present; (iii) the final phase involving clear clinical manifestations of significant cardiac disease and impairment concluding with end-stage HF and premature death. Thus, during childhood through to the early teenage years, DAC is usually asymptomatic in both DMD and BMD, despite autonomic-induced sinus tachycardia, which increases cardiac activity to compensate for respiratory fatigue [189], displayed in approximately 26% of cases [190]. Electrocardiogram abnormalities are affected by morphological changes in both thin and thick cardiac muscle filaments [191]. During a transitional phase, the posterobasal left ventricle and its lateral wall are enlarged by subendocardial fibro-fatty replacement and cardiomyocyte hypertrophy, causing the initial diastolic dysfunction and Q-wave irregularities. This process continues to eccentric hypertrophy and continued worsening of cardiac function [185]. Cardiac fibrosis is related to cardiac muscle decline, and it appears in 17% of patients under 10 years, 34% between 10 and 15 years, and 59% of 15-year-olds and older [192], although the rates vary depending on the patient cohort examined and the imaging modality used to detect the fibrosis. Sinus tachycardia increases with disease duration, as well as the degree of systolic dysfunction and ventricular arrhythmias, due to the progressive nature of cardiomyocyte atrophy and subsequent ventricle and atrial dilation and thinning of the ventricular walls [185]. Heart-rate variability affects about 51% of MD patients and it is strictly correlated to arrhythmias and fibrosis for the BMD phenotype, while DMD cases are often arrhythmic even in the absence of cardiac fibrosis [193]. Dilated cardiomyopathy is the final clinical stage and ultimately advances to HF. This may depend on telomere length, which correlates with the likelihood of developing dilated cardiomyopathy [194].

### 3.5. Current Therapeutic Approaches for Muscular Dystrophy

The clinical management and treatment of MD has progressed from predominately monothematic, i.e., neurology, to the current multidisciplinary approach involving cardiologists, neurologists, pulmonary specialists and respiratory therapists, anesthesiologists, orthopedists, psychologists, physiotherapists, physical therapists, occupational therapists, speech therapists, dietitians, genetic counselors, allied health assistants, and social workers. However, the complexity and the level of care received by people with MD depend on a number of variables such as socioeconomic factors and the specific treatment center frequented. DMD and BMD remain terminal illnesses, although the improvements in patient care and disease management have decelerated disease progression. Current therapies are limited to symptom management; however, new therapies targeting absent or nonfunctional dystrophin are under development or have already reached the clinic. We give a brief overview of the principal therapies for MD.

#### 3.5.1. Pharmacotherapies Targeting Secondary Effects

The majority of the drugs prescribed to MD patients are principally limited to the management of symptoms that target pathological pathways activated by the loss of dystrophin. These pathways include inflammation, calcium homeostasis, ROS production, fibrosis, functional cardiac ischemia, and impaired muscle regeneration. Corticosteroids are the main drugs used to ameliorate skeletal muscle disease by decreasing inflammation in favor of repairing damaged myocyte membranes and prolonging the endogenous repair strategies of resident skeletal progenitor cells. However, despite also increasing respiratory function and reducing cardiac involvement (4% for each year of treatment) [195], corticosteroids have several serious side effects, e.g., Cushing’s syndrome, growth failure and short stature, delay of puberty, hypertension, hyperglycemia, weight gain, cataracts, and osteoporosis, which temper enthusiasm for this treatment option, particularly for long-term use [196]. In addition, other molecules already used to ameliorate cardiovascular symptoms such as angiotensin-converting enzyme (ACE) inhibitors and beta-blockers can target impaired cardiac activity in a manner that does not address the underlying cause of DAC. Diuretics are often combined with ACE inhibitors as they remove extra water and salt from the blood that further ameliorate cardiac workload and blood pressure [197].

Many commercially available drugs may benefit MD patients, and some are being tested on animal models such as muscular dystrophy (*mdx*) mice, and preclinical precision models provide a solid basis for clinical trials in both DMD and BMD patients. Among these, the synthetic copolymer poloxamer P188 stabilizes the sarcolemma and improves cardiac phenotype [198], the phosphodiesterase 5 (PDE5) inhibitor, sildenafil, improves NO signaling [199], losartan reduces fibrosis by decreasing TGF-β expression [198], and the combination of nonsteroidal anti-inflammatory drugs and NO donors maintains cardiac function and reduces systemic inflammatory markers [200].

Newer therapies, targeting different pathways altered in MD patients, are at a developmental phase and others have already reached clinical trials [201,202,203]. For example, idebenone, a free-radical scavenger, passed phase I/II trials after showing some beneficial effects for DMD patients [204,205,206]. A confirmatory phase III randomized placebo-controlled trial demonstrated an amelioration in the respiratory function of DMD patients receiving idebenone [207,208]. A recent long-term data analysis supported the disease-modifying treatment effect of idebenone previously observed in randomized controlled trials [209]. Another treatment approach involves the use of inhibitors of myostatin, a member of the TGF-β superfamily that negatively regulates muscle growth [201,202]. Different types of molecules have been tested including antibodies; however, these treatments demonstrated no to moderate effects in clinical trials [201,202,203]. For example, histone deacetylase (HDAC) inhibitors, by altering the epigenetic markers of histones, were demonstrated to influence the expression of multiple genes including some involved in adverse cardiac remodeling [210]. Thus, it was proposed that HDAC inhibitors could activate the expression of antifibrotic, anti-inflammatory, and proregenerative genes. The HDAC inhibitor givinostat was used in phase I/II clinical trials in ambulatory DMD patients and demonstrated improvements as assessed by histological analyses of muscle biopsy material [211]. Currently, a phase III randomized, double-blind, placebo-controlled trial in DMD patients is ongoing.

#### 3.5.2. New and Experimental Therapies Targeting the Primary Defect

In addition to these symptomatic therapies, new treatments that address the direct cause of MD are currently under investigation in preclinical trials with positive results that show how these new generation of therapeutics have the potential to ameliorate pathology and function loss in both the skeletal muscle and the heart.

One of the approaches tested to ameliorate the symptoms of MD patients is the upregulation of utrophin, a homolog of dystrophin that shares 80% of its sequence and acts similarly to dystrophin by binding the DGC and determining the length of F-actin filaments during muscle development [212,213,214]. It has been hypothesized that utrophin upregulation could partially compensate for the lack of dystrophin. Many molecules were evaluated in preclinical studies such as ezutromid (SMT C1100) that increased utrophin mRNA and protein levels in the *mdx* mouse model [215]. Clinical trials were performed but significant effects are appreciable only at high doses because utrophin cannot completely compensate for the absence of dystrophin [216].

Since nonsense mutations are frequent in DMD patients, another strategy tested was the use of compounds able to promote ribosomal read-through of premature stop codons, allowing the continuation of translation and the production of the protein [217,218]. Early clinical trials showed inconclusive and contradictory results [219]. However, more recent trials that used the ribosomal read-through drug ataluren highlighted that some patient subgroups were positively affected by the treatment [220]. Additional clinical trials or precision model investigations need to be performed in order to verify clear clinical efficacy and which subgroups of MD patients stand to benefit from read-through therapy.

Another drug targeting *DMD* transcription/translation are antisense oligonucleotides (AONs) that are short single-stranded DNAs able to induce exon skipping. AONs allow one or more exons to be excluded (or “skipped”) which restores the reading frame and enables the production of a partially functional, albeit shorter, dystrophin protein. AONs are capable of treating 83% of DMD patients (79% with a deletion, 91% with small mutations, and 73% with a duplication) [221]. Different AON chemical modifications have been developed, and two of these, the phosphorodiamidate morpholino (eteplirsen) and the 2′OMe (drisapersen), were used in clinical trials involving males with DMD. Both of these types of AONs target exon 51 and induce frame-restored dystrophin production following a single intramuscular injection [222,223]. In two trials (phase II), the group assigned to receive drisapersen reached the clinical endpoint [224]. However, a larger phase III study did not demonstrate clinical benefit. Moreover, a recent meta-analysis of five randomized controlled trials (RCT) of eteplirsen and drisapersen showed no significant overall effect for exon-skipping treatments [225]. The use of these molecules for the treatment of DMD patients still remains controversial. Next-generation AONs are in development in order to improve their delivery and efficacy, e.g., adding short peptide sequences called “cell-penetrating peptides” or novel stereochemical modifications of the 2’OMe backbone proved to be positive alterations [226,227].

Gene therapy with the replacement of the mutant *DMD* gene with a WT version would be the only way to totally “cure” MD patients. However, at the moment, the large size of the *DMD* gene (2.2 Mb) and its transcript mRNA (14 kb) precludes incorporation in any known vectors. In addition, the majority of viral vectors do not transfect with high efficiency in cardiac muscles. On the contrary, most serotypes of adeno-associated virus (AAV) show muscle tropism; however, these viruses are very small and possess modest transgene holding capacity (~4.5 kb) [228]. To overcome this limit, mini- or microdystrophins were developed on the basis of observations in BMD patients who express partially functional dystrophin protein variants, which contain deletions of the central rod domain, yet are mildly affected relative to DMD patients [228,229]. Presently, three independent clinical trials using different microdystrophins, AAV vectors, and gene promoters are in progress for DMD. Preliminary results of these ongoing trials show that systemic delivery of AAV to skeletal muscle is now feasible and results in high amounts of microdystrophin expression in treated DMD patients’ muscles. Nevertheless, some questions and challenges remain open, including how functional microdystrophin will be in humans, how long the transgene will be expressed, the type of preparation of viral vectors, systemic delivery capability, the scale necessary to treat patients, and the final timeline and treatment costs [201].

The development of genome editing technologies has opened up new horizons to restore the production of dystrophin protein in DMD patients and, among these, CRISPR/Cas9 is the most promising [201,230,231]. This technology is widely used in vitro to induce or to revert mutations in targeted regions and could also be applied to the *DMD* gene. However, there are some problems and challenges for therapeutic use of CRISPR/Cas9 in DMD patients. Indeed, as mentioned earlier, HDR is active only in proliferating cells and is, thus, not an option in post-mitotic cells such as cardiomyocytes. Moreover, the majority of patients carry exon deletions that cannot be easily repaired using CRISPR/Cas9. Nevertheless, NHEJ can still be used to restore the *DMD* reading frame, e.g., by deleting an exon. Additionally, by editing specific *DMD* sequences, splicing sequences can be modified/disrupted, causing exon skipping in a similar way to AONs, or they can also be used to delete one or more exons. Both strategies allow reading-frame restoration with the production of a BMD-like dystrophin. Since genome editing approaches act on DNA, all mRNA produced from the edited *DMD* gene will be corrected, thus avoiding the necessity of repeat treatments. Many different studies evaluated the efficacy and safety of the genome editing using CRISPR/Cas9 as a therapy for DMD with varying rates of success (for a complete overview, we refer the reader to the review of Lim et al. (2018) [230]). Translation to the clinic for the direct treatment of DMD patients is currently limited due to different problems including in vivo delivery strategy, immune response activation, and off-target effects. Another option to treat MD patients could be in vitro editing of the *DMD* gene in patient-specific iPSCs followed by differentiation into specific cells and autologous transplantation.

The aim of cell-based therapy, in the context of MD, is to introduce dystrophin-expressing cells derived either from healthy precursor cells or from genetically modified patients’ cells, which are capable of tissue integration and repopulation of injured muscles with functional cells. Cells are normally delivered to individual muscles via intramuscular injection which is an often painstakingly slow and inefficient approach. However, implanted cells undergo long-term self-renewal and minimal immunological activation [232]. Therefore, although inefficient, this approach is still under intense investigation. The main precursor cells used in preclinical studies and clinical trials for MD cell therapy are satellite cells [233,234], muscle-derived stem cells [235,236,237,238,239], myoblasts [240,241,242], pericytes [243], bone-marrow-derived stem cells [235,244,245], cluster of designation (CD) 133^+^ stem cells [246,247], and mesangioblasts [248,249,250]. Finally, another interesting approach tested on *mdx* mice is the use of cardiosphere-derived cells (CDCs) which are progenitor cells intrinsic to the heart [251]. Aminzadeh and coworkers demonstrated that treatment of *mdx* mice with CDCs improves cardiac and skeletal myopathy, and positively augments cardiac function, ambulatory capacity, and survival [251]. In addition, exosomes obtained from human CDCs induce similar beneficial effects in *mdx* mice and DMD patient-specific iPSC-CMs [251]. A testament to the translatability of this research group’s approach constitutes the results of the halt cardiomyopathy progression in Duchenne “HOPE” randomized, open-label, interventional phase I/II clinical trial (NCT02485938) of intracoronary delivery of an allogeneic CDC cell-therapy product (CAP-1002, Capricor Therapeutics™, Beverly Hills CA, USA) in 25 DMD patients (males >12 years of age, genetically diagnosed with DMD, with significant cardiac scarring). Participants receiving standard of care and the cell-therapy product showed sustained improvement of upper-limb activity, significant scar reduction with improved inferior wall systolic thickening, and ameliorated pulmonary functions [252]. Moreover, on the basis of this success, the HOPE-2 trial (NCT03406780) also tested CAP-1002; however, this time, the product was delivered intravenously every 3 months to males >10 years of age at a more advanced stage of disease compared to the first HOPE trial. The recently completed HOPE-2 trial showed impressive results for patients, and the outcomes of negotiations with regulators is expected to yield positive results for final approval.

Taking a forward and wider look into the future of DAC therapy, the development of iPSCs and genome editing technologies has opened up the possibility to correct *DMD* mutations in patient-specific iPSCs followed by their differentiation into muscle precursor cells or cardiomyocytes that can be transplanted into MD patients [162,201,253]. Furthermore, the generation of bespoke genome-edited patient-specific iPSC cell lines could also be a valuable asset to the cardiac disease modeling tool kit.

## 4. Modeling Dystrophy-Associated Cardiomyopathy

As described previously, dystrophin-associated cardiomyopathy (DAC) is the leading cause of death in MD patients. This cardiomyopathy shows great heterogeneity in terms of onset, severity, and progression rate, and knowledge about the pathological mechanisms is very limited. Furthermore, there are no prognostic biomarkers or strong genotype–phenotype correlations. For these reasons, the development of precision medicine models that recapitulate in vitro the pathomechanisms and phenotypes observed in MD patients is pivotal to find better treatments. Historically, the only possibility to model DAC was to use animal models; however, they do not accurately recapitulate the human disease course. The discovery of iPSCs and their ability to differentiate into cardiomyocytes paved the way to create an in vitro model of this aspect of MD pathology. iPSC-CMs have the potential to replace damaged patients’ CMs and offer a scalable theranostic platform for personalized DAC modeling useful for studies correlating cellular and clinical phenotype determinants, and screening potential therapeutic approaches.

### 4.1. Animal Models

Previously, the only way to study and understand the pathobiology of dystrophin deficiency and to develop therapies for treating MD was based on the use of animal models. Currently, there are more than 60 different animal models available, ranging from small nonmammalian to large porcine models. [15,154,254]. Homology in the dystrophin gene has been found in both vertebrates and invertebrates, which have a high level of sequence similarity. This high conservation allowed the development of different models starting from nonmammalian ones such as *Caenorhabditis elegans*, *Drosophila melanogaster*, and *Danio rerio*, to mammalian models including mice, rat, rabbit, cat, dog, and pig [15,154,254,255,256]. Although animal models are important tools in MD research, they present limitations because they do not always faithfully recapitulate MD human pathophysiology. All the currently available models present specific peculiarities with different advantages and disadvantages that we briefly report below.

Despite nonmammalian models possessing different musculature and certain MD pathologies with respect to mammalian MD, they do have some advantages such as physiological simplicity and the ease of genetic manipulation [256]. 

*C. elegans* possesses a dystrophin gene ortholog called *dys-1* and a multiprotein complex similar to the DGC complex [257,258]. Since *C*. *elegans* is easily manipulated, various mutant strains with nonsense mutations in different *dys-1* gene positions have been generated [259]. *C. elegans* carrying such mutations showed widespread degeneration of corporeal wall muscles. *Dys-1*-mutated *C. elegans* were employed for high-throughput genetic studies and to screen different molecules that act on a wide spectrum of targets [260,261]. Recently, two different strains were developed and used to isolate and sequence muscle-specific transcriptomes at different stages of the disease progression [262]. This analysis reveals that the absence of dystrophin leads to broad splicing errors and that two different groups of genes contribute to the dystrophic phenotype. In particular, the first gene set is activated early in development and disease progression and comprises genes involved in mitochondrial function, cell death and protein degradation signaling in muscle. The second group of genes is activated in the final half of the developmental cycle, maintained through adulthood, and it is associated with the establishment and maintenance of muscle structure [262]. However, although *C. elegans* is important for genetic screening strategies, the musculature of C. *elegans* is starkly different from that of mammals and, hence, ultimately fails to recapitulate full MD pathology [154].

*Drosophila melanogaster* has been widely used to characterize and model different human diseases, including MD [263]. The *D. melanogaster* dystrophin gene (*Dys*) is present as different isoforms similar to humans. Moreover, genes encoding DGC proteins are highly conserved [256,263] in this model. To recapitulate MD, different mutations in *Dys* were generated in *D. melanogaster* that show mobility defects, age-dependent muscle degeneration, and neuronal and circulatory defects [263,264,265]. This model was also used to screen different phenotype ameliorating compounds [266].

*Danio rerio*, also known as zebrafish, is another nonmammalian organism used as model for many different human diseases. Zebrafish present abundant skeletal muscle and express almost all the DGC proteins, including dystrophin, with a similar localization observed in humans. Many different dystrophin-deficient zebrafish were identified and also obtained using morpholino knockdown. These mutants showed extensive muscle degeneration, fibrosis, inflammation, and necrosis [267,268]. These models have been widely applied to study exon-skipping therapies, demonstrating that, to rescue a severe phenotype, 20–30% of normal dystrophin levels are needed [267,268].

With regard to mammalian MD models, a broad range of them were developed and used in research. Although more complex with respect to nonmammalian models, they do have the advantage of being more similar to humans. Mouse models are widely used in MD research fields and, among these, the dystrophic *mdx* mouse is the best known and used model. This dystrophic mouse, discovered in the 1980s, carries a nonsense mutation in exon 23 of the murine *Dmd* gene that blocks the production of full-length dystrophin [269,270]. Despite their extensive use, *mdx* mice do not faithfully recapitulate the human phenotype, which is particularly evident for DAC. In addition, *mdx* skeletal muscle disease is milder than that observed in DMD patients (with the exception of the diaphragm), and the lifespan is only reduced by about 25% [271,272,273]. The most severe DMD symptoms, including muscle wasting, scoliosis, cardiomyopathy, and heart failure, appear only in older mice (15 months or older) or purposefully stressed mice, e.g., *mdx* mice challenged with isoproterenol [274,275,276,277,278,279,280]. The *mdx* model has been crossed with various genetic backgrounds such as albino, BALB/c, FVB, and immune-deficient murine strains, and different phenotypes were observed (for a complete review, see McGreevy et al. (2015) [15]). To better recapitulate the human dystrophic phenotype, mice models have been modified by eliminating compensatory mechanisms or by humanizing strategies. Among these, one of the most studied is the utrophin/dystrophin double-knockout mouse [281,282]. These mice appear smaller with respect to single *Dmd* null-mice and have a more severe muscle phenotype, similar to/worse than that observed in humans. Other studies showed that utrophin heterozygous *mdx* mice could be a better intermediate model between the severe utrophin/dystrophin double-knockout and the milder *mdx* mice [283,284,285]. However, in contrast to humans, these double-knockout mice carry two mutations: one in the *Dmd* gene and the other in the *Utr* gene. How this second alteration influences data interpretation and meaningfulness is not entirely agreed upon.

Using genome-engineering techniques, two different strain of rats, with mutations in exon 23 or deletions between exons 3 and 16 of the *Dmd* gene, were developed and analyzed [286,287]. Rats with the exon 23 mutation showed severe fibrosis in muscles, with additional adipocyte infiltration, muscle weakness and decreased activity. Moreover, they developed cardiomyopathy characterized by eccentric hypertrophy and altered diastolic dysfunction [286]. The other rat model with deletions between exons 3 and 16 showed dystrophic pathology and, additionally, these rats presented greater cardiac fibrosis at an earlier stage but there were no clear indications of functional cardiomyopathy [287].

Recently, a rabbit model generated by CRISPR/Cas9 editing of exon 51 to abrogate dystrophin expression was described [288]. The majority of these *DMD* KO rabbits died prematurely; however, surviving rabbits showed typical DMD histological defects in skeletal muscles and developed cardiomyopathy with decreased left-ventricular ejection fraction and fractional shortening [288].

Larger DMD models were developed in cats, dogs, and pigs [15,154,254]. Although dystrophin-deficient cats were characterized, this MD model is not used since the mutant cats developed tongue hypertrophy that made feeding and drinking difficult and, hence, it was not ethically sensible to use them as an MD model [289].

Many different breeds of dogs were reported with dystrophin-deficient MD, and many of these were selected to establish DMD canine models. Generally speaking, the canine phenotype is more severe with respect to that observed in *mdx* mice. Thus, canines are considered a better model of human DMD [15,290]. The first and most extensively used canine MD model is the golden retriever with muscular dystrophy (GRMD), which carries a splice site mutation in intron 6 that causes exon 7 skipping and a lack of dystrophin protein production [290,291]. Another important model is the beagle X-linked muscular dystrophy, in which the GRMD mutation is crossed on a beagle background [292,293]. The clinical course and progression of the disease in affected dogs is similar to that observed in humans. In particular, the first year of the GRMD model is very similar to the first 20 years of a DMD patient’s life [294]. For example, by around 2–3 months of age, GRMDs present limb weakness and exercise intolerance, while, at 6 months, atrophy of muscles, joint contractures, dysphagia, hypersalivation, and cardiac involvement can be observed, and death generally occurs at approximately 3 years of age. Furthermore, from a histological point of view, the observed muscle lesions are similar to that of human MD including fewer myofibers with central nucleation and fibrosis in limb muscles and the heart [15,295]. Since cardiomyopathy and congestive heart failure are clearly evident and measurable in MD canine models, they are considered to be the best model to study the cardiac aspects of MD [295]. However, there is also some divergence between affected dogs and humans, e.g., higher mortality rates at birth (20–30%) [293,296,297,298], ambulation maintained in young affected dogs [293,299], disease progression stabilizing at 6–10 months, and observations of increased phenotype divergence [293,298,300]. Taken together, DMD canine models present many similarities to DMD patients, making them an interesting model to analyze human MD pathomechanisms and for preclinical therapeutic studies including gene therapies [299].

Lastly, pigs are promising animal models for different human diseases as they are comparable to humans in terms of size, musculature, diet, and immune and cardiovascular systems. Dystrophin-deficient pigs carrying an exon 52 deletion were developed [301]. These animals showed clinical similarity to humans with MD including the absence of dystrophin expression in skeletal muscle, interstitial fibrosis, presence of mononuclear inflammatory cells, increased serum creatine kinase levels, muscle weakness, and reduced mobility [301]. The major problem is reduced lifespan (≤3 months) that precludes natural breeding and, therefore, dramatically impacts the uptake of this model [301]. However, the porcine MD model was recently used to test and validate somatic gene editing techniques that aimed to restore the *DMD* reading frame [302]. Intramuscular injection of AAV carrying Cas9 and a pair of guide RNAs targeting sequences flanking exon 51 into pigs with an exon 52 deletion restored the production of a shortened dystrophin protein and improved skeletal muscle function. Furthermore, systemic application of the same AAV vector caused widespread dystrophin expression, including in the diaphragm and heart, which prolonged survival and reduced arrhythmias [302]. Similar results were also obtained following genome editing of iPSCs derived from a DMD patient who had an exon 52 deletion. Skeletal and cardiac myocytes differentiated from this patient’s corrected iPSCs showed ameliorated skeletal myotube formation, cardiomyocyte Ca^2+^ handling, and arrhythmia susceptibility [302]. This is a good example of a translational study in which a suitable animal model is combined with in vitro experiments using patient-derived cells.

### 4.2. iPSC-Based Models

Conventionally, in addition to animal models, the in vitro study of disease pathomechanisms can be applied, involving different approaches such as the use of primary or immortalized cell lines; however, these methods have many limitations. Particularly pressing issues for DAC are that disease-specific cell types are not always readily accessible and most primary cells cannot indefinitely proliferate and be maintained in long-term culture. On the other hand, immortalized cells can be easily cultured but do not always accurately reproduce the physiological condition. The first step to obtain an iPSC-based model of a particular disease is to reprogram person-specific somatic cells followed by the differentiation of the obtained iPSCs into various disease-relevant cell types. e.g., cardiomyocytes or neurons, which are then used to identify pathological molecular mechanisms. To model DAC, iPSCs have to be differentiated into the cells present in the heart, i.e., not only cardiomyocytes, but also stromal, endothelial, and immune cells, and afferent/efferent neurons. Different studies were published in which iPSC-derived cells were used to model DMD pathology. Here, we first describe the methods and problems encountered in cardiac differentiation of iPSCs and follow up by giving a brief summary of the principal studies using iPSC-based models of DAC undertaken to date.

#### 4.2.1. Derivation of Cardiomyocytes from iPSCs

Both types of PSCs (embryonic and induced) were used to generate cardiomyocytes [303,304,305]. The first differentiation protocols were based on the coculture of PSCs with mouse visceral endoderm-like (END-2) stromal cells that produce signaling molecules to drive cardiac induction, and on differentiated embryoid bodies (EB) which mimic early embryonic development through the combination of physical and chemical cues to direct PSCs into spontaneously beating cardiomyocytes [306,307]. However, these two methods have limited utility as shown by a very low yield of beating cardiomyocytes, immature phenotype, and low success rates. For these reasons, different PSC monolayer differentiation protocols were developed that mostly relied on modulating signaling pathways guiding embryonic development, e.g., Activin/Nodal/TGF-β, GSK3/Wnt, and BMP, together with undefined factors supplied by mouse embryonic fibroblast-conditioned media or contained in media supplements [308]. Lian and collaborators developed a frequently used method on the basis of the temporal modulation of the Wnt/β-catenin pathway that generated yields of cardiomyocytes above 90% [309]. Partial and direct reprogramming are two novel strategies for the differentiation of fibroblasts into cardiomyocytes without going through an iPSC stage. However, this method is not frequently used in biomedical research due to low efficiency rates (only 20% of obtained cells are cardiomyocytes) [310,311].

Despite the development of many CM differentiation protocols, the efficiency is variable, influenced by different factors including cell density and cell-cycle state [312].

#### 4.2.2. Concerns about iPSC-CMs

iPSC-CMs are an essential source for genetic and morphological characterization of patient-specific cells, and they allow the in-depth study of many molecular mechanisms which appear to be deregulated during the progression of DAC. However, even once iPSCs are successfully differentiated into cardiomyocytes, there are some issues that must be taken into account, including the immature state of cardiomyocytes and the heterogeneity of the obtained cell populations.

Cardiomyocytes derived from both ESCs and iPSCs appear significantly structurally and functionally immature compared to human adult cardiomyocytes. PSC-derived cardiomyocytes show a fetal-like phenotype, which is an important issue because the immature phenotype could affect their capacity to recapitulate the physiology and pathology of adult cells, e.g., their response to drugs. Changes in many different cardiomyocyte properties such as cell morphology (shape, size), contractility (sarcomere organization, myosin light chain, and troponin isoform expression), gene expression, electrophysiology (ion-channel expression and localization, cell–cell coupling, conduction velocity), calcium handling (transport by SERCA), and metabolism (mitochondrial maturity and energy source flexibility) [313,314] are associated with increased cardiomyocyte maturation.

With regard to morphology, iPSC-CMs show morphological similarities to early fetal cardiomyocytes; indeed, both types of cardiomyocyte have a rounded shape and a single nucleus [313,315,316]. During maturation, cardiomyocytes adopt an elongated morphology, become binucleated, and, at the end stage, mature adult cardiomyocytes are rod-shaped with about 33% being binucleated [313]. In addition, adult cardiomyocytes are anisotropic, which facilitates electrical conduction and contractility. Conversely, iPSC-CMs are randomly aligned and develop spontaneous electrical activity [313,316].

There are considerable differences in the contractile machinery between iPSC-CMs and adult cardiomyocytes. Sarcomeres in particular are less organized in iPSC-CMs compared to late-stage fetal cardiomyocytes. Adult mature cardiomyocytes display highly aligned sarcomeres uniformly distributed along the cell and contain high densities of aligned myofibril structures. On the other hand, iPSC-CMs are similar to early fetal cardiomyocytes with randomly aligned and distributed sarcomeres, predominantly localized in the perinuclear region. They present less dense myofibril structures [313,316,317,318]. Normally, mature sarcomeres show striations that are assembled from overlapping and non-overlapping myofilaments. Mature cardiomyocytes show all the prominent myofilament regions such as: I-bands, A-bands, M-lines, and H-zones, in addition to the Z-discs [313,319]. Sarcomere striations in iPSC-CMs are generally not well defined, with only Z-discs and sometimes I-bands with immature unorganized sarcomeres being visible. However, the majority of contractile proteins present in adult cardiomyocytes can be found in iPSC-CMs, although they are expressed at lower levels and/or are present in different isoforms [315].

There are consistent differences in electrophysiology between iPSC-CMs and adult mature cardiomyocytes. Indeed, iPSC-CMs spontaneously contract asynchronously as a consequence of their immature electrical coupling, whereas mature cardiomyocytes are excited when an electrical stimulus is delivered and they contract synchronously [313]. The cardiac action potential (AP) can be divided into four different phases and is a consequence of the involvement of different ion channels that act in a time-specific manner. Analogous to the structural machinery, ion channels change along with cardiac developmental maturation, resulting in varying AP profiles. Due to their immature phenotype, iPSC-CMs lack a majority of inward rectifying potassium channels (KCNJ2) that are present in mature cardiomyocytes, while they possess pacemaker current channels normally absent or poorly expressed in adult ventricular cardiomyocytes [313,314,320,321,322]. The spontaneous contractility of iPSC-CMs is a consequence of pacemaker channels along with unstable resting membrane potentials [315]. There are also differences in the expression of calcium channels, particularly T-type channels, normally detected in cardiac conducting cells but not in mature ventricular cardiomyocytes, which are present in various amounts in iPSC-CMs. On the contrary, L-type calcium channels present in mature cardiomyocytes are expressed at low levels similar to that observed in fetal cardiomyocytes [313,322,323]. In addition to the different expression of ion channels, cell–cell connections between iPSC-CMs are also different with respect to mature cardiomyocytes and influence their electrophysiological function. Indeed, the spatiotemporal distribution of the proteins involved in cell–cell connection or communication, e.g., connexin-43, N-cadherin, and cardiac sodium channel NaV1.5, change during development and maturation of cardiomyocytes. Cell–cell connection proteins are sporadically distributed along the plasma membrane of iPSC-derived and fetal cardiomyocytes and display no distinguishable intercalated discs that contribute to the immature asynchronous beating [316,324]. The immature electrophysiology of iPSC-CMs is similar to that observed in fetal cardiomyocytes and impacts many parameters dependent on electrical efficiency. In particular, depolarization velocity, capacitance, and conduction velocity all appear lower in AP traces from iPSC-CMs compared to mature cardiomyocytes [313,315,316,317,325,326,327].

The structures involved in calcium handling also mature during development and, unsurprisingly, iPSC-CMs show immature calcium-handling apparatus. In particular, they do not form transverse tubules, which results in reduced calcium-handling efficiency and impaired excitation–contraction coupling. iPSC-CMs also have an underdeveloped SR with lower amounts of proteins like SERCA, RyR, and calsequestrin that are involved in physiological calcium cycling during contraction [313,328,329,330].

With regard to metabolism, adult cardiomyocytes show a mature network of mitochondria that occupy 35% of the cell volume and are aligned to the sarcomeric direction, which is necessary to provide energy for contraction [331,332]. Conversely, iPSC-CMs display immature mitochondria that appear smaller, disorganized, without the presence of cristae, and localized in the perinuclear region [313,333]. In adult cardiomyocytes, the majority of energy is produced through oxidative metabolism, whereas iPSC-CMs principally use the less efficient glycolytic energy production pathway [313,333].

During the physiological developmental program, mechanical and electrical stimuli and the interaction with ECM and noncardiomyocytes synergistically combine to drive the maturation of cardiomyocytes. In order to increase the maturity of iPSC-CMs different approaches that mimic the physiological environmental have been used. In particular, in in vitro mechanical and/or electrical stimulation, ECM interactions and cocultures with noncardiomyocyte cells have been applied to increase the maturation of iPSC-CMs. However, even with these methods, iPSC-CMs did not acquire fully mature phenotypes [314,316].

Along with the immature phenotype, another important issue is the heterogeneity of the resultant differentiated populations of iPSC-CMs. Indeed, all the developed cardiac differentiation protocols lead to a mixed population of cardiomyocytes and noncardiomyocytes of various proportions. The impact of these non-myocytes on iPSC-CM properties is still controversial. For example, in one study, it was reported that, when non-myocytes were removed during differentiation, the maturation of iPSC-CMs was slower; in particular, the electrophysiology and the calcium-handling functions were altered. Interestingly, when nonmyocyte cells were added back to early isolated cardiomyocytes, they rescued the block of electrophysiological maturation, suggesting that noncardiomyocytes are important for electrophysiological maturation [334]. Moreover, there is also heterogeneity between obtained cardiomyocytes that result in different properties associated with atrial, ventricular, and/or nodal/pacemaker subtypes. Additionally, it was demonstrated that cardiac differentiation produces a mixed population of these cardiomyocyte subtypes that have specific molecular and functional properties [335].

To overcome the problem of heterogeneous cell populations, many different approaches were developed to purify and enrich a specific cardiomyocyte population. The more frequently used methods are antibody-based selection through fluorescence-activated cell sorting (FACS) or magnetic-activated cell sorting. Both methods distinguish desired/undesired cells via the expression of particular cell-surface markers, e.g., vascular cell adhesion molecule 1 [336,337]. However, it is important to consider that these protein markers are not completely specific or selective. Other approaches use metabolic differences between cardiomyocytes and nonmyocytes, e.g., cardiomyocyte yields can be enriched using glucose-depleted media supplemented with lactate [338]. On the basis of this approach, it was demonstrated that, using media without glucose and glutamine supplemented with lactate, it is possible to eliminate the iPSCs remaining after differentiation [339]. Other approaches including molecular beacons to tag cardiomyocyte-specific mRNA or microfluidic systems are in the initial stages of development but may prove useful in future applications [340].

#### 4.2.3. DAC Models using iPSC-CMs

In the last few years, iPSC-CMs have been widely used to model cardiac diseases including DAC, and more than 20 original articles reported the use of iPSC-derived cells from MD patients. Starting from the first iPSC line derived from skin fibroblasts of a DMD patient in 2008 [341], numerous other iPSC cell lines derived from different somatic-cell sources obtained from DMD and BMD patients were generated and published [133,253,302,342,343,344,345,346,347,348,349,350,351,352,353,354,355,356,357,358,359,360,361,362,363,364,365,366,367,368]. These works demonstrated that it is possible to use iPSC-derived cells carrying specific *DMD* mutations to reproduce MD pathogenesis and to verify new therapeutic approaches. The principal studies focusing on DAC are reported in Table 1. Early studies aimed to demonstrate that MD patient-specific iPSCs could be differentiated into functional iPSC-CMs that recapitulated the clinical phenotype observed. Moreover, the use of iPSC-CMs allowed the discovery of a variety of pathogenic mechanisms caused by the absence of dystrophin. Among them, calcium handling is one of the most studied in iPSC-CMs. Indeed, the use of different DMD iPSC-CMs permitted the demonstration that calcium homeostasis is altered in DMD iPSC-CMs, which showed slower Ca^2+^ transients [342], profound reduction of the L-type calcium current with augmented cytosolic Ca^2+^ levels [344], increased diastolic Ca^2+^levels and Ca^2+^transient amplitudes after a short-term mechanical stretch protocol [360], augmented intracellular diastolic Ca^2+^levels [355], slowed Ca^2+^transient rise, and decay compared to controls after field stimulation pacing [363]. In addition to calcium handling, alterations in iPSC-CM DMD models showed increased levels of ROS [366], overexpression of immunoproteasome subunits, and increased release of tumor necrosis factor (TNF)-α and cTnI [355].

In addition to the study of DAC molecular mechanisms, the iPSC-CM platform has been widely used to test potential therapeutic strategies and, among these, genetic therapies were the most studied. Indeed, many works using iPSC-CMs demonstrated the amelioration of defects observed in DMD iPSC-CMs after restoring a truncated form of dystrophin achieved through exon skipping or delivery of micro- or minidystrophin [302,350,353,359,365]. Lin and colleagues demonstrated that treatment with the membrane sealant compound poloxamer 188 significantly decreased resting cytosolic Ca^2+^ level, repressed caspase-3 activation, and consequently suppressed apoptosis in DMD iPSC-CMs [344]. Additionally, the immunoproteasome inhibitor ONX-0914 was shown to improve the health of iPSC-CMs as indicated by reduced sarcolemmal damage and proinflammatory signaling in MD iPSC-CMs treated with ONX-0914 [355]. In another study, Afzal and collaborators showed that the vasodilator nicorandil protected iPSC-CMs from damage observed as a consequence of *DMD* mutations, i.e., reduced DNA damage and mitochondrial stress [346]. These studies demonstrate the beneficial effects of these compounds that could pave the way for clinical trials.

Taken together, these results demonstrated that iPSC-CMs recapitulate the major DAC phenotypes and play an important role in the development of new therapies. However, there are some limitations of the studies carried out to date. In particular, as previously reported, one of the major drawbacks of most iPSC-CM-based cardiomyopathy models is the variable maturity of the iPSC-CMs used, a critical point which is not always analyzed in every study and one that could have a big impact on the observed disease phenotypes. Moreover, the genetic background is not always considered, and the data obtained from patient-derived iPSC-CMs were generally compared to healthy donors’ iPSC-CMs. Only a minority of reports used genome-edited isogenic controls that would diminish the influence of comparing disease readouts to “controls” with different genetic backgrounds. Another shortcoming of these approaches to modeling DAC relates to cellular aspects such as the predominant use of monocellular cardiomyocentric culture that negates the role of other cell types involved in DAC pathological mechanisms, e.g., stromal, neuronal and immune cells. Notably, the elegant work of Chang and colleagues [369] analyzed both iPSC-CMs and iPSC-vascular smooth muscle cells (VSMCs) and observed telomere shortening specifically in iPSC-CMs and not in iPSC-VSMCs, which suggests a role for mechanical stress in CM-specific telomere erosion due to the lack of dystrophin. Lastly, although several studies corroborated findings in animal models and patients’ explanted heart tissue, to the best of our knowledge, the Chang et al. [369] study is the only report that analyzed measurable parameters from patients’ heart tissues and matched them to those observed in iPSC-derived cardiovascular cells. In the coming years, the fast-paced nature of precision medical research will likely see the rapid adoption of these more nuanced advanced patient-specific cell models of DAC.

### 4.3. Studying Disease in Multicellular Models

The heart is a heterogeneous environment composed of ECM and different cell types that do not work independently but communicate together through finely regulated crosstalk. This intercellular communication plays an essential role in maintaining homeostasis and in regeneration and remodeling of damaged tissue during pathophysiological processes. Cardiomyocytes represent only 30% of the entire cardiac cell population; the balance includes smooth muscle cells and endothelial cells of the coronary vasculature and endocardium, cardiac fibroblasts, and immune cells (both resident and recruited from the systemic circulation). A specific example of the cell-to-cell crosstalk can be observed in the endothelium, where endothelial cells can release NO, angiotensin II (Ang II), prostaglandins, pro/anticoagulant factors, and various growth factors including fibroblast growth factor (FGF) and vascular endothelial growth factor (VEGF), which impact various myocardial and vascular functional parameters, e.g., endothelial cells regulate heart size following angiogenesis [370,371]. In addition, endothelium cells induce cardiac hypertrophy in order to support the heart during hemodynamic stress. For instance, endothelium-induced VEGF overexpression and activation of the MAPK cascade by neuregulin 1-release leads to cardiac hypertrophy [372,373]. Cardiac fibroblasts (cFb) are fundamental for the structural and mechanical maintenance of the myocardium by coordinating ECM production and remodeling to ensure scar formation following tissue damage. Initially, this process serves to favor correct electrical conduction and maintain rhythmicity of cardiac electrical stimuli. However, in the presence of biochemical stresses such as overproduction of Ang II, FGF-2, and insulin growth factor I, cFbs increase ECM protein release and alter myocyte contractility, oxygenation, and metabolism. Moreover, in the pathological environment, cFbs are stimulated to differentiate into myofibroblasts that can express smooth-muscle contractile proteins, proliferate rapidly, and secrete significant amounts of ECM leading to cardiac fibrosis. This fibrotic loop is exacerbated by the production of TGF-β, which, as well as being responsible for cardiac hypertrophy by activating the SMAD signaling pathway in iPSC-CMs [355], also induces epithelial to mesenchymal transformation of endothelial cells into cFbs, thus increasing the pool of cells secreting ECM [374]. These cell types could play an important role in the development of the DAC. There are different protocols that describe the production of endothelial cells and cFbs from iPSCs. Among them, Giacomelli and coworkers developed a protocol to efficiently obtain cFBs and endothelial cells and to coculture these cells with iPSC-CMs in three dimensions (3D) [137]. Moreover, they also demonstrated that the coculture of CMs, cFbs, and cardiac endothelial cells, all obtained from iPSCs, enhanced the maturation of iPSC-CMs, thus opening new possibility in the model of cardiac disease.

Immune system components are involved in cardiac repair and regeneration, and they are responsible for triggering numerous processes including damage-associated signaling, inflammation, revascularization, dedifferentiation and cell replacement, and fibrotic scar formation/resolution. Immediately after an injury, apoptotic and necrotic cells release ROS, hydrolases, proteases, and damage-associated molecular patterns, which stimulate pattern recognition receptors both on resident cells such as endothelial cells and resident macrophages and on circulating innate immune cells such as neutrophils, monocytes, and dendritic cells that are recruited to the site of damage. Moreover, cFbs and innate immune resident cells can also mediate and propagate this mechanism through chemokine and cytokine production. Innate immune cells in the heart modulate cardiac pathophysiology by infiltrating the injured area, clearing cell debris, and promoting inflammation and resolution, tissue restoration by secreting growth factors, and ECM remodeling via the activation of matrix metalloproteinases [375]. The role of the adaptive immune system in cardiac repair and regeneration is less known. B cells are associated with autoimmunity against healthy cardiomyocytes after cardiac injury, while T cells play versatile roles in autoreactivity, inflammation modulation, and tuning macrophage polarity [376,377,378,379]. Among immune cells, the heart possesses specific resident macrophages that are in direct contact with cardiomyocytes and endothelial cells. These cardiac resident macrophages perform many roles and are active in the steady state and after a disruption of homeostasis. Under normal conditions, resident cardiac macrophages have an anti-inflammatory phenotype, which is indicated by the expression of CD45, CD11b, and F4/80 surface markers, with MHCII^hi^ (major histocompatibility complex II), MHCII^low^, Ly6c^+^, and CCR2^−^ cells also included in this heterogeneous population of cells. Only a small percentage of macrophages, with Ly6c^hi^ and CCR2^+^ immuno-phenotype, resident in the myocardium are derived from blood monocytes [380]. In the healthy heart, macrophages regulate angiogenesis, ECM turnover, capillary density, and collagen production. Macrophages also guard against infection and influence cardiomyocyte activity, e.g., cardiac macrophages facilitate electrical conduction by connecting to cardiomyocytes through connexin 43, which induces a more positive resting potential and accelerates cardiac repolarization [381]. Following cardiac injury, resident macrophages expand without the input of blood-derived monocytes. MHCII^hi^ cell populations efficiently process and present antigens to T cells, which suggests an immune surveillance role for cardiac macrophages. On the other hand, a subset of MHCII^low^ macrophages can phagocytose dying cardiomyocytes, and they contribute to local homeostatic processes by clearing potentially harmful cell debris. [380]. Some research groups developed protocols to differentiate iPSCs into specialized macrophages. Takata et al. in 2017 demonstrated the ability of iPSCs to differentiate into primitive-like macrophages that acquired a microglia-like pattern of gene and protein expression when cocultured with neurons, suggesting that growth factors and organ-specific cues promote terminal macrophage differentiation [382]. Cao and coworkers developed a protocol that combines the use of serum-free medium and time-dependent addition of specific growth factors to efficiently induce iPSC differentiation into cells that resemble yolk sac-derived erythro-myeloid progenitors (EMPs) expressing CD43 and CD45 surface markers. In turn, EMPs were shown to be able to generate monocytes and M0 macrophages that can be polarized to proinflammatory M1 macrophages and anti-inflammatory M2 macrophages [383].

Given the importance of non-CM cells in the pathophysiology of the heart, modeling cardiac diseases is moving from single-cell iPSC-based models to multicellular models that contain, in addition to iPSC-derived cells, other important cell types such as cFbs, endothelial cells, and immune cells. Another source of modeling improvement derives from the generation of 3D tissue models that were shown to better recapitulate the pathogenic situation compared to two-dimensional (2D) models [137]. Combined, these two aspects also have a significant impact on the maturation of iPSC-CMs. Indeed, different studies demonstrated that iPSC-CM maturation is improved by the presence of other cell types and 3D structure provided by the ECM [137,345,384,385,386,387,388]. Although, to date, these advanced 3D multicellular models have not been applied to MD patients’ iPSCs, they do stand a greater ability to recapitulate DAC phenotypes more faithfully and with better resolution. These new advances may boost the discovery of new disease-altered pathways exploitable for development of novel DAC-specific treatments.

## 5. Concluding Remarks

The development of cardiac models that recapitulate the phenotypes observed in MD patients is fundamental to understand underlying pathomechanisms and find new therapies. Animal models developed so far have been widely used to shed light on different aspects of MD pathology; however, they do not fully recapitulate the human cardiac phenotypes. Since their discovery, iPSCs were applied to disease modeling and drug screening platforms. iPSCs can be considered the best cells for precision medicine since they carry a patient-specific genome and can theoretically give rise to any cell type present in the human body: a powerful combination of factors for use in disease phenotype analyses. On this premise, iPSC-CMs have shown enormous potential to model DAC and perform drug screening in a patient-specific manner. The continuous increase in the number of publications featuring DAC models using iPSC-CMs is evidence that the employment of these cells benefits the future outlook of DAC research. iPSC-CMs can recapitulate patient-specific phenotypes in vitro and identify new DAC pathomechanisms and drug target options. Nonetheless, some issues make it difficult to fully recapitulate patients’ phenotypes including iPSC cell-line variability, efficiency of differentiation protocols, immature phenotype of iPSC-derived cells, and use of monocellular models. However, these challenges are being overcome by new technology such as genome editing and multisystem organoid models of mammalian physiology. In the near future, DAC modeling will be centered on assembling multicellular 3D models that contain all disease-relevant cell types or all cells functionally affected by *DMD* mutations. For this to become a reality, the continued close collaboration among patients, family members and friends, clinicians, allied health workers, researchers, advocacy groups, and funding bodies is of fundamental importance and should never be underestimated or undervalued. Returning to the opening quotation and in the context of this review’s subject matter, it can be said that continued refinement and innovation of iPSC-based DAC models will ensure that researchers’ goals are not thwarted.

## Figures and Tables

**Table 1:** Principal studies using cardiomyocytes derived from induced pluripotent stem cells (iPSC-CMs) to model and study dystrophin-associated cardiomyopathy (DAC).

*DMD* Mutation	Aim of the Work	Readout	Ref.
Deletion of exons 48–50; Deletion of exons 47–50; Nonsense c.10171 C > T in exon 70; Deletion of two nucleotides in exon 35.	Restore the expression of dystrophin in iPSC-CMs using antisense oligonucleotides (AONs) to mediate skipping of exon 51, or viral vectors containing a dystrophin minigene.	iPSCs from seven patients were obtained and differentiated into iPSC-CMs. The mutations caused altered dystrophin expression in all iPSC-CMs. AONs and dystrophin minigene restored dystrophin expression to up to 90% of normal levels.	[365]
Deletion of exon 50.	Generation and characterization of DMD iPSC-CMs from iPSCs generated from urine derived shed epithelial cells.	Duchenne’s muscular dystrophy (DMD) iPSC-CMs showed a specific phenotype that was different from normal iPSC-CMs, e.g., increased membrane susceptibility to hypotonic stress, slower Ca^2+^ transients in the early phases after differentiation mPTP opening, and altered cell metabolism.	[342]
Deletion of exons 4–43.	Investigate whether a human artificial chromosome (HAC) carrying the whole *DMD* genomic sequence inserted into muscular dystrophy (MD) iPSCs could induce and maintain the expression of different dystrophin isoforms during cardiomyocyte differentiation.	iPSC-CMs from healthy, DMD, and corrected DMD cell lines were analyzed at day 24 of differentiation for dystrophin protein expression. HAC-modified DMD iPSC-CMs and healthy iPSC-CMs expressed the longer dystrophin isoforms, i.e., Dp427 and Dp140, in addition to the short form of dystrophin (Dp71).	[343]
Deletion of exons 45–54. Deletion of exons 46–47.	Establish iPSCs from two DMD patients, differentiation into iPS-CMs followed by characterization.	T-lymphocytes from two DMD patients were reprogrammed into iPSCs and differentiated into contracting iPSC-CMs that expressed cardiac proteins with the exclusion of dystrophin.	[347]
Deletion of exons 42–52.	Study the molecular mechanisms underlying dilated cardiomyopathy in DMD iPSC-CMs and screen the therapeutic effectiveness of the membrane sealant poloxamer 188 (P188).	DMD iPSC-CMs displayed dystrophin protein deficiency, elevated resting Ca^2+^, mitochondrial damage, and apoptosis. Treatment with P188 significantly decreased the resting cytosolic Ca^2+^ level, repressed caspase-3 activation, and consequently suppressed apoptosis. Whole-transcriptome sequencing between DMD iPSC-CMs and control iPSC-CMs detected significant gene expression changes in genes linked to apoptosis, contractility, and heart diseases.	[344]
Out of frame deletion of exons 3–6. In frame deletion of exons 45–53.	Investigate the effects of vasodilatory drug, nicorandil, on iPSC-CMs carrying deletions in the *DMD* gene and in *mdx* mice.	DMD iPSC-CMs showed decreased levels of eNOS (nitric oxide synthase) and nNOS, increased cell injury and cell death after 2 h of stress and recovery. This was associated with increased levels of reactive oxygen species (ROS) and dissipation of the mitochondrial membrane potential. Healthy and DMD iPSC-CMs treated with nicorandil showed decreased cellular stress compared to nontreated DMD iPSC-CMs. Inhibition of cyclic guanosine monophosphate-nitric oxide (cGMP-NO) signaling or adenosine triphosphate (ATP)-sensitive potassium channels abrogated the protective effects of nicorandil.	[346]
* Out of frame deletion of exons 49–50. * In frame deletion of exons 45–55.	Determine the contribution of Nup153 to the epigenetic alterations that occur in DAC using animal models, human primary tissue, and MD iPSC-CMs.	Following NO deregulation, Nup153 protein expression was significantly increased in *mdx* hearts compared with controls. Nup153, regulated by acetylation, was recruited to chromatin where it regulated cardiac remodeling gene transcription, e.g., the actin-binding protein nexilin. In DMD and BMD iPSC-CMs, Nup153 protein expression and intracellular localization were altered. In addition, upregulated acetylated Nup153 was also found in heart tissue from DMD patients.	[368]
Deletion of exons 48–50.	Restore dystrophin expression in patient-derived iPSC-CMs using clustered regularly interspaced short palindromic repeats (CRISPR)-Cpf1.	Dystrophin expression was restored through exon skipping or exon reframing, in iPSCs derived from a DMD patient. Corrected iPSC-CMs expressed a truncated dystrophin isoform that ameliorated the phenotype, enhancing the contractile function and respiratory capacity of mitochondria.	[350]
Deletion of exon 50.	Evaluation the effects of cardiosphere-derived cells (CDCs) both in *mdx* mice and in DMD iPSC-CMs.	Treatment of iPSC-CMs with CDC-exosomes suppressed beat-to-beat calcium transient alterations during pacing and improved cell metabolism.	[251]
Deletion of c.3638–3650 c.6599 C > G. Deletion of c.9204–9207.	Verify the link between the length of telomeres and mutations in cardiac contractile proteins using iPSC-CMs.	Telomere length was reduced in DMD iPSC-CMs compared with vascular smooth muscle cells differentiated from the same iPSC line and healthy iPSC-CMs.	[369]
Deletion of exons 3–6.	Investigate the effects of exosomes in dystrophin-deficient iPSC-CMs.	Healthy and DMD exosomes protected iPSC-CMs from stress-induced injury by decreasing ROS and also delayed mPTP opening that led to decreased cell death. These cardioprotective effects were dependent on the presence of exosomal surface proteins and activation of ERK1/2 (extracellular signal-regulated kinases 1/2) and p38 MAPK (mitogen-activated protein kinase) signaling.	[366]
Deletion of exons 48–50. Point c.6913–4037 T > G. Duplication of exons 55–59.	Correction of different *DMD* mutations in MD iPSCs and functional assays to test the impact that these various mutations have on iPSC-CMs.	Dystrophin expression was recovered using CRISPR/Cas9 in all three iPSC lines generated. iPSC-CMs derived from corrected iPSCs showed improved contraction forces despite only 50% of iPSC-CMs being corrected.	[353]
Male: nonsense c.5899 C > T. Female: deletion of exons 8–12.	Investigate electrophysiological dysregulation in iPSC-CMs from a male patient and a manifesting female carrying a *DMD* mutation. Analyses of X Chromosome reactivation (XCR), inactivation (XCI), or erosion (XCE) following cell reprogramming and cardiac differentiation.	XCR or XCE were observed during reprogramming and cardiac differentiation of both dystrophin-deleted and female fibroblasts. Female iPSC-CMs coexpressed wild type (WT) and deficient levels of dystrophin protein. Funny current (*I_f_*) voltage-dependent calcium channel activity was altered in male and female iPSC-CMs. Beat-rate variability was also demonstrated in male and female iPSC-CMs.	[367]
Deletion of exons 49–50.	Investigate the role of the immunoproteasome (IP) and IP inhibitor ONX-0914 in dystrophic cardiomyopathy using the *mdx* mouse model and patient-derived iPSC-CMs.	MD iPSC-CMs showed increased intracellular Ca^2+^ and increased cTnI and tumor necrosis factor (TNF)-α release compared to healthy iPSC-CMs. The specific IP subunits, PSMB8 and PSMB9, were upregulated in MD iPSC-CMs. The IP inhibitor ONX-0914 reduced intracellular Ca^2+^ concentration, release of cTnI and TNF-α, and the expression of collagen 3a and transforming growth factor (TGF)-β in MD iPSC-CMs. Similar results were found in heart tissue from *mdx* mice treated with ONX-0914.	[355]
Deletion of exons 46–55.	Verify the beneficial effects of exon 45-skipping.	The treatment of iPSC-CMs with phosphorodiamidate morpholino oligomers targeting exon 45 restored the production of dystrophin with beneficial effects on Ca^2+^ homeostasis that attenuated arrhythmic events.	[359]
Deletion of exon 44.	Analyze the consequences of the *DMD* mutation on Ca^2+^ handling in iPSC-CMs.	Patient-specific iPSC-CMs showed increased _i_Ca^2+^ compared to healthy iPSC-CMs. Mechanical stretching increased _i_Ca^2+^ in DMD iPSC-CMs but not in control iPSC-CMs.	[360]
Deletion of exons 3–7. Deletion of exons 4–43. Genome edited deletion of exons 3/4.	Model DMD cardiomyopathy using DMD patient-specific iPSC-CMs and identify physiological changes and future drug therapy targets.	DMD iPSC-CMs had significantly increased arrhythmic calcium traces compared to isogenic control iPSC-CMs that were significantly decreased with propranolol treatment. Moreover, fibrotic genes were upregulated in dystrophin *null* iPSC-CMs, which also had similar dysregulated molecular programs and biological processes to those observed in *mdx* hearts and human left ventricular (LV) samples from DMD patients.	[362]
Deletion of exon 52.	Evaluation of the effects of genome editing experiments in porcine and iPSC models of DMD.	The induced expression of a shortened dystrophin protein in skeletal and cardiac myocytes through exon 51 skipping improved skeletal muscle function, reduced arrhythmogenic vulnerability, and prolonged survival. Patient-derived iPSCs were genetically edited with exon 51-skipping AONs and differentiated into myoblasts and cardiomyoblasts. iPSC-CMs showed ameliorated Ca^2+^ handling and arrhythmogenic susceptibility.	[302]
Deletion of exon 50. Genome edited point mutation c.263delG.	Analyze the role of dystrophin in CM development and cardiomyopathy.	iPSC-CMs displayed a lack of full-length dystrophin protein expression, reduced myofibril contractile tension, delayed relaxation kinetics, and altered Ca^2+^ homeostasis. Moreover, the absence of dystrophin led to retarded or altered maturation of iPSC-CMs.	[363]

* These mutations were identified subsequently to the publication referenced in the table. Further details on the identification of the mutations can be found in Spaltro et al., *Stem Cell Res*, 2017 and Gowran et al., *Stem Cell Res*, 2018.

## Abbreviations

AAV	adeno-associated virus
ACE	angiotensin-converting enzyme
Ang II	angiotensin II
AONs	antisense oligonucleotides
BMD	Becker’s muscular dystrophy
BMP	bone morphogenetic proteins
CamKII	calmodulin calcium-dependent kinase II
CD	cluster of designation
CDC	cardiosphere-derived cells
cFb	cardiac fibroblasts
CK	creatine kinase
CM	cardiomyocytes
CRISPR/Cas9	clustered regularly interspaced short palindromic repeats/CRISPR-associated protein 9
cTnI	cardiac troponin I
DAC	dystrophin-associated cardiomyopathy
DGC	dystrophin glycoprotein complex
DMD	Duchenne muscular dystrophin
DMD	Duchenne’s muscular dystrophy
DSB	double-stranded DNA breaks
EB	embryoid bodies
ECM	extracellular matrix
EMPs	erythro-myeloid progenitors
ERK1/2	extracellular signal-regulated kinases 1/2
ESCs	embryonic stem cells
FACS	fluorescence-activated cell sorting
FGF	fibroblast growth factor
GRMD	golden retriever with muscular dystrophy
HAC	human artificial chromosome
HDAC	histone deacetylase
HDR	homology-directed repair
HF	heart failure
IP	immunoproteasome
iPSC	induced pluripotent stem cell
iPSC-CMs	cardiomyocytes derived from iPSCs
LV	left ventricular
MAPK	mitogen-activated protein kinase
MD	muscular dystrophy
MET	mesenchymal-to-epithelial transition
MHC	major histocompatibility complex
mPTP	mitochondrial permeability transition pore
NF-κB	nuclear factor kappa-light-chain-enhancer of activated B cells
NHEJ	non-homologous end-joining
iNOS	inducible nitric oxide synthase
nNOS	neuronal nitric oxide synthase
ORF	open reading frame
PI3K	phosphoinositide 3-kinase
PDE5	phosphodiesterase 5
PSCs	pluripotent stem cell
QC	quality control
Rac1	Ras-related C3 botulinum toxin substrate 1
ROS	reactive oxygen species
RyRs	ryanodine receptors
SERCA	sarco-endoplasmic reticulum calcium ATPase
SMAD	smaller mothers against decapentaplegic
SR	sarcoplasmic reticulum
TGF-β	transforming growth factor-beta
TNF-α	tumor necrosis factor
TRPC	transient receptor potential cation
VEGF	vascular endothelial growth factor
VSMCs	vascular smooth muscle cells
WT	wild type
XCE	X chromosome erosion
XCI	X chromosome inactivation
XCR	X chromosome reactivation
